# EphrinA2 Receptor (EphA2) Is an Invasion and Intracellular Signaling Receptor for *Chlamydia trachomatis*

**DOI:** 10.1371/journal.ppat.1004846

**Published:** 2015-04-23

**Authors:** Prema Subbarayal, Karthika Karunakaran, Ann-Cathrin Winkler, Marion Rother, Erik Gonzalez, Thomas F. Meyer, Thomas Rudel

**Affiliations:** 1 Department of Microbiology, Biocenter, University of Wuerzburg, Wuerzburg, Germany; 2 Department of Molecular Biology, Max Planck Institute for Infection Biology, Berlin, Germany; 3 Steinbeis Innovation gGmbH, Center for Systems Biomedicine, Stuttgart, Germany; Purdue University, UNITED STATES

## Abstract

The obligate intracellular bacterium *Chlamydia trachomatis* invades into host cells to replicate inside a membrane-bound vacuole called inclusion. Multiple different host proteins are recruited to the inclusion and are functionally modulated to support chlamydial development. Invaded and replicating *Chlamydia* induces a long-lasting activation of the PI3 kinase signaling pathway that is required for efficient replication. We identified the cell surface tyrosine kinase EphrinA2 receptor (EphA2) as a chlamydial adherence and invasion receptor that induces PI3 kinase (PI3K) activation, promoting chlamydial replication. Interfering with binding of *C*. *trachomatis* serovar L2 (*Ctr*) to EphA2, downregulation of EphA2 expression or inhibition of EphA2 activity significantly reduced *Ctr* infection. *Ctr* interacts with and activates EphA2 on the cell surface resulting in *Ctr* and receptor internalization. During chlamydial replication, EphA2 remains active accumulating around the inclusion and interacts with the p85 regulatory subunit of PI3K to support the activation of the PI3K/Akt signaling pathway that is required for normal chlamydial development. Overexpression of full length EphA2, but not the mutant form lacking the intracellular cytoplasmic domain, enhanced PI3K activation and *Ctr* infection. Despite the depletion of EphA2 from the cell surface, *Ctr* infection induces upregulation of EphA2 through the activation of the ERK pathway, which keeps the infected cell in an apoptosis-resistant state. The significance of EphA2 as an entry and intracellular signaling receptor was also observed with the urogenital *C*. *trachomatis*-serovar D. Our findings provide the first evidence for a host cell surface receptor that is exploited for invasion as well as for receptor-mediated intracellular signaling to facilitate chlamydial replication. In addition, the engagement of a cell surface receptor at the inclusion membrane is a new mechanism by which *Chlamydia* subverts the host cell and induces apoptosis resistance.

## Author Summary

*Chlamydia trachomatis* are major human pathogens causing ocular and sexually transmitted diseases with hundreds of millions of cases per year. *Chlamydia* replicate inside the host cell in a membrane bound vacuole called inclusion. The current concept on how *Chlamydia* communicates with the host cell during its replication is based on the identification of the host protein that interacts with *Chlamydia*. Here, we describe that *C*. *trachomatis-*serovar L2 and D use EphA2, a member of the largest class of human receptor tyrosine kinases, as an adherence and entry receptor that is endocytosed together with the bacteria. Cell surface EphA2 receptor is adopted by *Chlamydia* to function also at the inclusion to support growth and replication and to keep the infected cell in an apoptosis resistant state. Thus, we show that EphA2 is an undiscovered important surface and intracellular signaling receptor that is crucial for chlamydial infection and development.

## Introduction

*Chlamydia trachomatis*, obligate intracellular human pathogens, cause a broad range of acute and chronic diseases in humans [1,2]. *C*. *trachomatis* serovars A-C are the cause of infectious blindness called trachoma, serovars D-K cause urinary or genital tract infections and serovars L1-L2 are associated with lymphogranuloma venereum (LGV). *C*. *pneumoniae* infections can result in community-acquired pneumonia and other respiratory infections. *C*. *trachomatis* has a biphasic developmental life cycle: infectious non-replicating elementary bodies (EB) are taken up by the host and transform into non-infectious reticulate bodies (RB) that reside and replicate inside a vacuole called inclusion. At the end of the cycle, RB differentiate back to EB that are released from the cell and start a new round of infection.

*C*. *trachomatis* entry into cells is a multifaceted process generally initiated by the interactions that occur between outer membrane proteins (OMPs) or type three secreted effectors, such as TARP with cell surface receptor and co-receptor molecules [3]. To maintain the life cycle inside the inclusion, *Chlamydia* are believed to secrete various effector proteins via their type three secretion system (T3SS), either into the host cell cytoplasm or through the inclusion membrane. Chlamydial inclusion membrane (Inc) proteins are integrated into the inclusion membrane and have been shown to interact with multiple host proteins and thereby manipulate the host cell [4]. *Chlamydia* is able to intercept trafficking vesicles via the recruitment of Rab proteins, which are able to specifically “snatch” vesicles from the retrograde intra-Golgi trafficking or interact with host organelles, such as the endoplasmic reticulum [5].

Host cell surface proteins including the estrogen receptor complex [6] and its subunit the protein disulfide isomerase (PDI) [6,7] are involved in *C*. *trachomatis* infection. For other chlamydial species, host cell receptor tyrosine kinases (RTKs) and their associated intracellular signaling cascades have been shown to play a pivotal role in facilitating adherence and regulating the infection. The platelet-derived growth factor receptor (PDGFR) is involved in *C*. *muridarum* uptake into non-phagocytic cells [8]. The fibroblast growth factor receptor (FGFR) is activated upon *C*. *muridarum* infection and is recruited to the cell surface associated EB [9]. The epidermal growth factor receptor (EGFR) serves as an invasion receptor for *C*. *pneumoniae* [10].

Several host signaling pathways have been shown to play an essential role for chlamydial development [11,12]. Signaling via PI3 kinase is required for normal development of *C*. *trachomatis* [13] and to keep the infected cell in an apoptosis resistant state [13,14]. To date, the mechanisms of how PI3 kinase is activated in *Chlamydia*-infected cells are not known.

Ephrin receptors and their membrane-bound ligands, Ephrins, constitute the largest known subfamily of RTKs, consisting of 16 receptors and 9 ligands that control important functions during development and adulthood (reviewed in [15]), including embryonic axon guidance, angiogenesis, cell death, migration and differentiation in development [16]. A unique feature of these receptors is their ability to undergo uni- and bidirectional signaling [17]. Forward signaling is mediated by the receptor-expressing cells upon ligand interaction, whereas reverse signaling is initiated by the ligand-expressing cell, both often mediating opposite effects. EphrinA2 receptor (EphA2) possess a highly conserved N-terminal glycosylated extracellular ligand-binding domain, a cysteine-rich region and two fibronectin type III repeats on the extracellular portion. The intracellular region of EphA2 is comprised of a juxtamembrane domain, a kinase domain, sterile alpha motif (SAM) and PDZ binding motif [18], which are essential to mediate protein-protein interactions. EphA2 and its glycosylphosphatidylinositol-linked ligand Ephrin-A1 act at the crosstalk between PI3K, MAPK, Src family kinases, Rho and Rac1 GTPases [19]. EphA2 is also considered to be an attractive therapeutic target against several human malignancies [20].

In the current study, we have identified EphA2 as a receptor for adherence and entry of *C*. *trachomatis*. Despite a rapid strong upregulation, EphA2 is depleted from the surface of infected cells and accumulates around the inclusion. EphA2 recruits and activates PI3 kinase, which is required for efficient replication of *Chlamydia*. Further, interference with EphA2 signaling affects normal infection and renders *Chlamydia*-infected cells sensitive to apoptosis induction. Thus, our results demonstrate that the cell surface receptor EphA2 is adopted by the chlamydial inclusion to support growth and replication.

## Materials and Methods

### Cell culture and bacterial infections

Human epithelial carcinoma cells (HeLa) (Source: ATCC no. CCL-2.1) were cultured in RPMI (GIBCO) with 10% Fetal Calf Serum (FCS) and primary uterine tube epithelial cells (Fimb) were cultured in RPMI (GIBCO) with 10% FCS on collagen coated plates. Human umbilical vein endothelial cells (HUVEC) (Source: Invitrogen) were cultured in medium-200 supplemented with low serum growth supplement (LSGS, Gibco BRL). Cells were grown at 37°C and 5% CO_2_. The propagation of *Ctr-*serovar-L2, *Ctr*-serovar D, *Ctr*-serovar A, *C*. *pneumoniae* and the recombinant *C*. *trachomatis* L2 strains [*Ctr*-pGFP::SW2, *Ctr*-pIncA-flag] in HeLa cells were made as previously described [21]. Stocks were stored frozen at -80°C in sucrose-phosphate-glutamate buffer [0.22 M sucrose, 10 mM Na_2_HPO_4_, 3.8 mM K_2_HPO_4_, 5 mM Glutamate, pH 7.4] and freshly thawed for each experiment. Cells were infected with bacteria in RPMI medium in the presence of 5% FCS and grown at 35°C with 5% CO_2_.

### Antibodies, inhibitors and recombinant proteins

Antibodies directed against EphA2 (WB analysis for total EphA2), *Chlamydia* Hsp60, GP96, PARP H-250, Pan Cadherin, GAPDH were purchased from Santa Cruz biotechnology. Antibodies against N-terminal EphA2 (D4A2) for WB and IF studies, pEphA2 (phospho EphA2 Ser897), pERK, pAkt, pPI3K, p85-PI3K, total Akt and total ERK were from Cell Signaling technology. Antibodies against N-terminal EphA2 (FACS, WB and IF), EphB4, PDGFRβ and IgG (control) were from R&D systems. Antibody against PDI was bought from Thermo Scientific. Anti-β-Actin and anti-Flag antibodies were purchased from Sigma-Aldrich. Phalloidin was bought from Invitrogen. Draq5 was from BioStatus Limited, UK. Polyclonal serum against IncA (anti-rabbit) was obtained through Gramsch laboratories. Inhibitors for PI3 kinase (LY294002), MEK1/2 (UO126) and EphA2 (dasatinib) were bought from Cell Signaling. Recombinant human EphA2 (rhEphA2) and recombinant human Ephrin-A1 homodimer (rhEphrin-A1 fused with IgG1-Fc) were purchased from R&D systems. Recombinant human prohibitin-His (rhPHB) also possess a His tag and is a control for rhEphA2. As a control for rhEphrin-A1, recombinant IgG1-Fc was bought from Life Technologies.

### Adherence assay

*Chlamydia* adherence assay was performed by pretreating the cells with rhEphrin-A1 (5 μg/ml) or with control for 1 h at 37°C followed by infection with purified EB for 1 h at 4°C in HBSS media (Life Technologies). EB was treated with rhEphA2 or with control (rhPHB) in a serum free media containing 0.1% BSA for 1 h at 37°C and the mixture was added to the cells and incubated for 1 h at 4°C. Cell surface EphA2 was blocked using control (IgG) or antibodies (10 μg/ml) against specific proteins for 1 h at 4°C followed by infection with EB for 1 h at 4°C. Before fixing the cells for immunostaining, cells were washed three times with PBS. Co-localization of EB with EphA2 was determined manually in a blinded study by quantifying from 8 different fields of higher magnification in 3 independent experiments.

### Pull-down assay

To prove the direct interaction of EphA2 with *Ctr*-EB, a pull-down assay was performed. *Ctr*-EB (L2) were purified as described before [22]. Purified *Ctr*-EB was incubated with rhEphA2 (10 μg/ml) or with rhEphrin-A1 (10 μg/ml) in a serum free RPMI media containing 0.1% BSA for 45 min at 37°C in a rotary shaker. Additionally, rhEphrin-A1-preincubated rhEphA2 was incubated with EB as above and the mixture was centrifuged at 5000 x g for 4 min at 4°C. The pellet was gently washed 3 times with ice cold-PBS by centrifugation at 5000 x g for 4 min at 4°C and subjected to western blot (WB) analysis.

### Invasion assay

*Chlamydia* invasion assay was performed by transfecting the cells with siRNA against luciferase gene (Luci) or EphA2 gene for 40 h at 37°C or treating the cells with IgG (control) or α-EphA2 antibody for 1 h at 4°C as mentioned in the adherence assay. The bacteria of required MOI were incubated with cells for 4–6 h at 35°C. The cells were fixed with 4% ice cold PFA for 30 min at 4°C. The fixed cells were taken for immunostaining after permeabilisation. Invasion assay in EphA2 overexpressed cells was performed after 40 h of EphA2-pcDNA3 transfection for flow cytometry analysis to determine the relative invasion rate of *Chlamydia* comparing to control empty-pcDNA3-transfected cells under permeabilised condition.

### Infectivity assay

Transfection and infection in HeLa cells were performed as described above. Supernatant from the primary infected cells was removed and the cells were washed with PBS. Cells from one set of experiment were lysed with glass beads and the lysate was passed through a pipette several times. The suspensions were transferred to HeLa cells (1:200 dilution) for secondary infection. The next day, cells were fixed with 4% PFA followed by immunostaining. Draq5 (DNA) was used for counterstaining of nuclei and Hsp60 antibody was used to stain the *Chlamydia* inclusion. Number of the inclusion was counted by analyzing on five random fields under the microscope and size of the inclusion was determined by ImageJ software (http://imagej.nih.gov/ij/).

### Generation of *Ctr* expressing recombinant-IncA (*Ctr*-pIncA-flag)

Full length IncA was fused with tag sequences (HA and Flag) by overlap PCR and inserted into pGFP::SW2 replacing GFP:CAT. The transformation of *Ctr* was performed as previously described [23]. The plasmid DNA (6 μg) and *Ctr* 1x10^7^ IFU were mixed in CaCl_2_ buffer (transfection mixture) and incubated for 30 min at room temperature (RT). The transfection mixture was added to McCoy cells and incubated for another 20 min followed by culturing in a T75 flask with fresh RPMI supplemented with 10% FCS. After 48 h, cells were lysed by sterile glass beads and the supernatant was used to infect the fresh cells which were cultured in the presence of 2 U/ml penicillin G and 2 μg/ml CHX and passaged every 48 h. When productive inclusion were visible, HeLa cells were used for infection and the concentration of penicillin was increased up to 50–70 U/ml to prepare the stock.

### Plasmids and site directed mutagenesis

An expression plasmid for EphA2 (accession NP_004422.2) was generated by PCR amplification using cDNA of pDONR223-EphA2 (Addgene ID:23926) [24]. The resulted amplicon was inserted into the pcDNA3 plasmid (Invitrogen). Mutant EphA2 without cytoplasmic domain (EphA2ΔIC) was created by inserting the region of human EphA2 encoding amino acids 1–560 into pcDNA3. EphA2 kinase dead mutant (EphA2K645R-plasmid) was obtained by PCR amplification using primers 5‘-ccggtggccatcaggacgctgaaagcc-3’ and 5‘-ggctttcagcgtcctgatggccaccgg-3’ which replaces Lysine to Arginine. Empty pcDNA3 was used as a control. p85-PI3K expression plasmid was purchased from Addgene (ID:11499) [25]. Transfection was performed using 1–1.5 μg/ml plasmid per well in 12 well plate using Polyethyleniminde (PEI) and OptiMEM transfection medium (Gibco). Transfection medium was changed after 5 h. Infection was performed at different time points after 20 h of transfection.

### siRNA and transfection

siRNA smart pool targeting EphA2 (siGENOME Human EphA2, 5 nmol, M-003116-02-0005) was purchased from Dharmacon. Pooled siRNA against PDI and PDGFRβ were bought from Santa Cruz. Cells at 60% confluent were transfected with siRNA using Lipofectamine 2000 (Invitrogen) and optimum. 8 h post transfection, medium was exchanged and 40 h post transfection, cells were infected with *Chlamydia* for different time points. siRNA targeting luciferase gene was used as a control, siLuci (Control): aacuuacgcugaguacuucga.

### Inhibitor assay

Cells were pre-treated with DMSO control or with dasatinib (DA) for 1 h at 37°C followed by infection with *Ctr*. Or 14 h infected cells were treated with the indicated concentrations of DA for 10 h. After 24 h of total infection, protein lysates were subjected to WB analysis or immunostaining. Uninfected or infected HUVEC cells were treated with DMSO or PI3K inhibitor LY294002 or MAPK inhibitor UO126 for 10 h. EphA2 plasmid-transfected HeLa cells were left uninfected or infected with *Ctr* for 24 h followed by treatment with DMSO or UO126.

### Flow cytometry (FACS)

For staining the surface exposed N-terminal EphA2, cells were grown in 6-well plates. After the respective time points of infection, cells were washed 3 times in ice cold PBS and detached using 5 mM EDTA at RT for 10–15 min. The cells were collected in Eppendorf tubes and washed 3 times in ice cold PBS containing 0.5% BSA (blocking buffer) by centrifugation at 350 x g for 4–5 min at 4°C. Cells were fixed with 4% paraformaldehyde (PFA) for 20 min at 4°C followed by incubation with 2% FCS for blocking to prevent unspecific binding of the antibody to the cell surface proteins. Antibody against EphA2 was diluted 1:200 in blocking solution and added to cells for 1 h at 4°C in the dark followed by a washing step in blocking buffer. Secondary anti mouse-Cy5 antibody (GE healthcare, 1:200) was incubated for 1 h followed by two washing steps in PBS and then taken for flow cytometry analysis. Via FSC-A and SSC-A, population of intact cells was determined and 10,000 events per sample were analyzed using “FACS AriaIII” and the “BD FACS Diva” software for all experiments. “FCSalyzer 0.9.3” software was used to create the overlay histograms. For surface EphA2, cells were not permeabilised whereas for detecting the total EphA2, cells were permeabilised using 0.2% Triton-X-100 in PBS. For analyzing the invasion rate of *Chlamydia* (invasion assay) in EphA2 overexpressing cells, *Ctr-*GFP was used. GFP was excited at 488 nm and Cy5 at 647 nm, respectively.

### Apoptosis sensitization assay

Transfection of the cells with siLuci or siEphA2 was performed as described above. Uninfected and *Chlamydia*-infected cells (MOI-1, 15–16 h) were induced to apoptosis with 50 ng/ml of human recombinant TNF-α (BD Pharmingen, San Diego, California) and 5 μg/ml of cycloheximide (CHX) from Sigma for 5–6 h. After the respective time post induction, the stimulated and control cells were fixed for immunostaining using TUNEL kit (Roche) or subjected to WB analysis to determine the PARP, cleaved PARP (C-PARP), Hsp60 and Actin.

### Re-infection assay

HeLa cells transfected with EphA2-pcDNA3 were first infected with *Ctr* (MOI-1) for 24 h followed by re-infection using EB (MOI-15) for 4 h. The cells were washed to remove the unbound EB and fixed for immunostaining against EphA2 (EphA2, red), *Ctr* (Hsp60, green) and Actin filaments (Phalloidin, blue). The total number of re-infected bacteria (both adherent and newly invaded EB) in EphA2 overexpressed and neighboring untransfected-infected cells were counted separately for quantification.

### Plasma membrane protein isolation

Plasma membrane proteins were isolated using the plasma membrane protein extraction kit (Abcam). Cells were grown in 150 cm^2^ dishes and infected for different time points. The cells were harvested, washed twice with ice cold PBS, resuspended in homogenization buffer and lysed with 30–50 strokes using a dounce homogenizer. 5% of the homogenate was collected as input. The homogenate was centrifuged to obtain total cellular membrane proteins in the pellet. The total cellular membrane proteins were subjected to further purification as described by the protocol of the supplier to obtain 10 to 100 μg of plasma membrane proteins in the pellet.

### TCA precipitation

Supernatants of the uninfected (UN) and time course infected samples were collected. To the samples, 250 μl of 72% TCA and 12.5 μl of 1.25% Na-deoxycholate was added to the final volume of 1 ml and incubated on ice for 30 min followed by centrifugation for 30 min at 14,000 x g in cold centrifuge. The pellet was washed with 400 μl of ice-cold acetone followed by another centrifugation for 10 min and then dried at 37°C for 5 min which was then resuspended by shaking for 15 min at 65°C followed by WB analysis.

### Immunofluorescence (IF) microscopy

The 4% PFA fixed cells were permeabilised using 0.2% Triton-X-100 for 30 min and blocked with 2% FCS for 1 h at RT. The cells were incubated with respective primary antibodies for 2 h at RT. After washing with PBS, cells were secondary stained with Phalloidin (Cy5) (Invitrogen) and Cy2 linked anti-mouse (Dianova) to stain the cell membrane and bacteria respectively for 1 h at RT. For staining the adhesive bacteria on the cell surface, the PFA fixed cells were blocked with 2% FCS and stained for extracellular bacteria without permeabilisation. Samples were washed as above and mounted with Mowiol (Roth). The exposure time was same for all images in each set of experiments.

### Immunoprecipitation and western blotting

For immunoprecipitation, UN or *Chlamydia*-infected cells were washed once with PBS and lysed in 1% NP-40, 150 mM NaCl, 2.5 mM EDTA/EGTA, 20 mM HEPES pH 7.4. The lysates were cleared by centrifugation at 20,000 x g and supernatants were incubated with α-EphA2 or α-p85-PI3K antibody for 1.5 h and precipitated using magnetic beads. All the steps and buffers used during immunoprecipitation were carried out at 4°C with phosphatase and protease inhibitor (Roche). The precipitated complexes were subjected to WB and were resolved by 8–12% sodium dodecyl sulfate (SDS)-polyacrylamide gel electrophoresis. Proteins were transferred to polyvinylidene difluoride membranes (Roche Diagnostics GmbH) and blocked with Tris-buffered saline containing 0.1% Tween 20 and 5% bovine serum albumin. Primary antibodies were incubated overnight at 4°C. Proteins were detected with peroxidase-coupled secondary antibodies using the ECL system (Pierce) and an Intas Chem HR 16–3200 reader. The signal bands were quantified by ImageJ software.

### Statistical analysis

For statistical calculations and histograms, Excel (Microsoft) was used. Statistical significance was calculated using the Student’s T test. Two-tailed T test was performed to calculate the P-value.

### Gene IDs

EPHA2 (1969), PDGFRβ (5159), EFNA1 (1942), EPHB4 (2050), PDI (64714), GP96 (7184), PHB (5245), TNF-α (7124), p85-PI3K (5290), AKT (207), PARP (142) and ERK (5594).

## Results

### EphA2 serves as a host cell receptor for *Ctr* adherence

The broadly accepted concept of the inclusion membrane as a signaling platform that orchestrates *Chlamydia*-host cell interaction prompted us to look for host cell proteins associated with the inclusion membrane using proteome analysis. The role of these proteins in chlamydial replication was analyzed further by RNA interference (RNAi) to identify host proteins that are required for chlamydial replication and development (MR, EG, PS, TR, TFM, in preparation). Unexpectedly, EphrinA2 receptor (EphA2), a cell surface RTK was among these proteins and was further investigated due to its potential presence in the *Ctr* inclusion proteome and its known role as a cell surface receptor.

**Fig 1:**
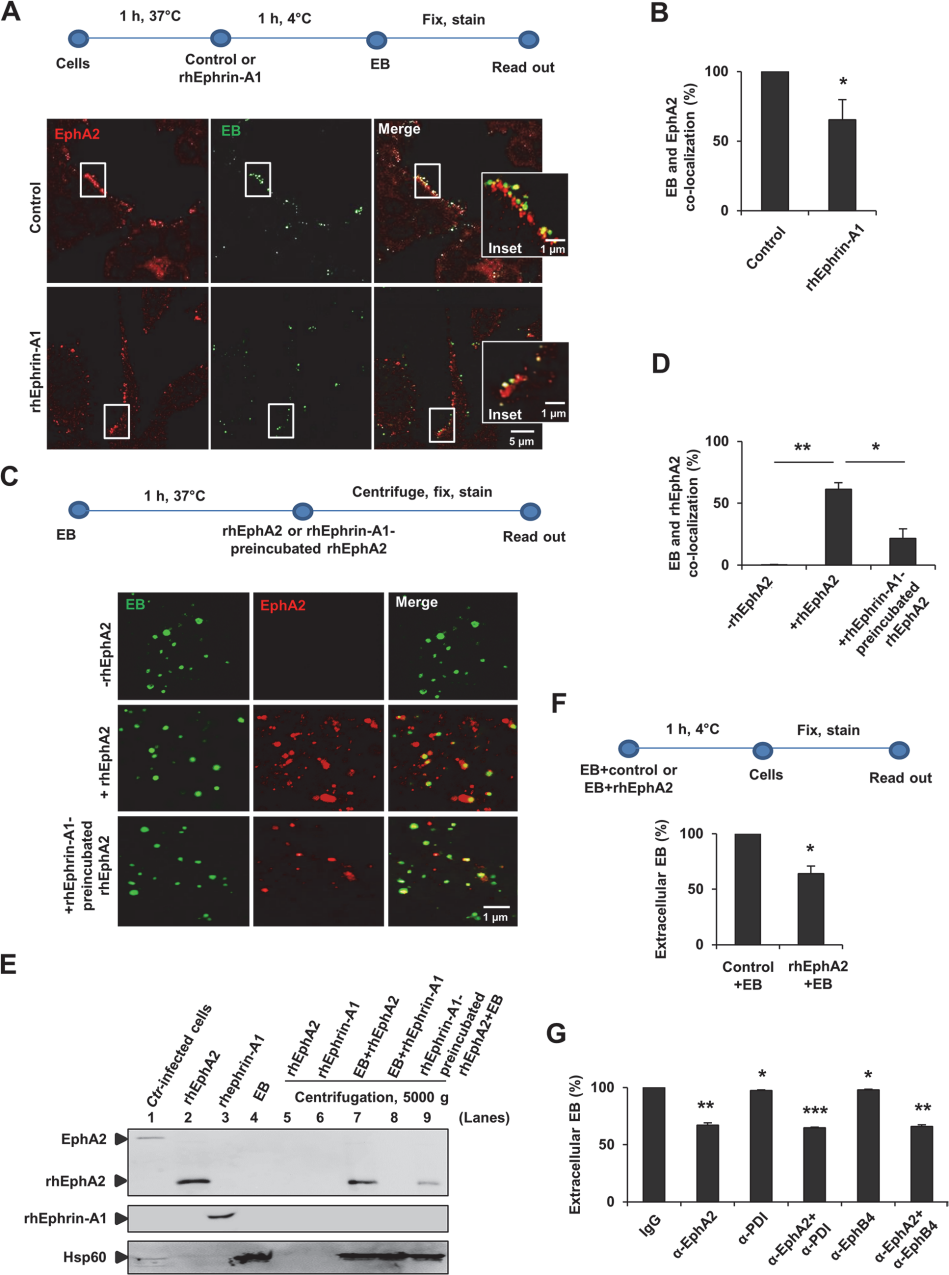
Interaction of EphA2 with *Ctr*-EB upon infection. (A, B) HeLa cells were pretreated with control or rhEphrin-A1 (5 μg/ml) for 1 h at 37°C and infected with *Ctr* (MOI-100) for 1 h at 4°C. Cells were fixed and immunostained for *Ctr* (Hsp60, green) and EphA2 (EphA2, red). (A) Images show EphA2 or EB or the co-localization of EB with EphA2 (yellow) on the cell surface. (B) Co-localization of EB with EphA2 was quantified from 8 different fields of view in 3 independent experiments. The data are expressed as a mean percentage of EphA2-associated EB (± SD) compared to total EB. *P<0.05. Error bars show mean ± SD. (C, D) Purified EB was incubated with or without rhEphA2 (5 μg/ml) in a serum free RPMI media containing 0.1% BSA for 1 h at 37°C. Additionally, rhEphrin-A1-preincubated rhEphA2 was incubated with EB as above. The mixture was centrifuged onto coverslips, washed 3 times with ice cold-PBS, stained and quantified as (B). The data are expressed as a mean percentage of EphA2-associated EB (± SD) compared to total EB. *P<0.05, **P<0.01. Error bars show mean ± SD. (E) Pull-down assay: Purified EB was incubated with rhEphA2 or with rhEphrin-A1 each 10 μg/ml in a serum free RPMI media containing 0.1% BSA for 45 min at 37°C in a rotary shaker. Additionally, rhEphrin-A1-preincubated rhEphA2 was incubated with EB as above and the mixture was centrifuged at 5000 x g for 4 min at 4°C. The pellet was gently washed 3 times with ice cold-PBS. *Ctr*-infected cells act as a positive control for full length EphA2 expression. The samples were subjected to WB analysis to determine the full length endogenous EphA2 (~130 kDa) and rhEphA2 (~70 kDa) using N-terminal EphA2 specific antibody. rhEphrin-A1 was expressed at ~55–60 kDa. Hsp60 acts as a control for EB. (F) EB pre-incubated with rhPHB (PHB-His) (PHB is a mitochondrial inner membrane protein. rhPHB acts as a control for His-tagged rhEphA2) or with rhEphA2 (EphA2-His) each 5 μg/ml for 1 h at 4°C were added to HeLa cells for 1 h at 4°C. Cells were fixed and immunostained for extracellular EB and Actin filaments (Phalloidin). The number of extracellular EB was counted randomly from 40 different cells. Data are expressed as percentage of extracellular EB relative to control. Shown is the mean ± SD of three independent experiments normalized to control+EB. *P<0.05. Error bars show mean ± SD. (G) HeLa cells were incubated with antibody against N-terminal-EphA2 or-PDI or-EphB4 each 10 μg/ml for 1 h at 4°C. Cells were then infected with GFP-expressing *Ctr* for 1 h at 4°C. Cells were fixed and immunostained against Actin filaments. Graph was made similar to (F). Shown is the mean ± SD of two independent experiments normalized to IgG control. *P<0.05, **P<0.01, ***P<0.001. Error bars show mean ± SD. (A, C) Magnification is indicated in size bar.

We first investigated if EphA2 can act as a cellular receptor for adherence of *Ctr* to host cells. Adherence assays were performed at 4°C to prevent uptake of adherent EB as previously described ([10]; see also Materials and Methods “Adherence assay” for details). We first tested whether EB co-localize with EphA2 and if Ephrin-A1, a known ligand of EphA2 may interfere with EB-EphA2 co-localization. EB co-localized with EphA2 and this co-localization was reduced by 35% upon pre-treating the cells with the recombinant human ligand rhEphrin-A1 (rhEphrin-A1; see Materials and Methods “Recombinant proteins” for details) (Fig 1A and 1B). These results suggested that EphA2 and EB interact on the surface of host cells in an Ephrin-A1-sensitive manner.

To further verify the direct interaction of EphA2 with EB, we incubated recombinant human EphA2 (rhEphA2) with isolated EB in the absence of host cells. EB and rhEphA2 co-localized in this cell-free assay and this co-localization was reduced when rhEphA2 was pre-incubated with the EphA2-ligand rhEphrin-A1 prior to incubation with EB (Fig 1C and 1D). Direct interaction was further confirmed in a pull-down experiment showing that purified EB interacts with purified rhEphA2 in a rhEphrin-A1-sensitive manner (Fig 1E). These experiments demonstrated a direct interaction of EphA2 with EB.

Pre-incubation of purified EB with purified rhEphA2 reduced the adherence of these EB to the cell surface by 36% compared to EB pre-treated with an unrelated His-tagged control protein (Fig 1F). These results suggested that EB adhere to epithelial cells via a bacterial ligand that interacts with EphA2, possibly via the ligand-binding domain of EphA2. Indeed, blocking the ligand-binding domain of EphA2 with an antibody directed against the N-terminus of EphA2 reduced adherence of EB to HeLa cells by 33% confirming a role of the ligand-binding domain in the EB-EphA2 interaction (Fig 1G). It was previously shown that blocking cell surface PDI using PDI antibody did not affect the *Ctr* attachment [26]. Therefore, as a known control, we blocked PDI and found no reduction in *Ctr* adherence (Fig 1G). Further, blocking an unrelated EphB4 receptor [27] had no effect on adherence of *Ctr* (Fig 1G).

### EphA2 is involved in host cell invasion of *Ctr*

We then investigated the role of EphA2 for the uptake of *Ctr* by HeLa cells (referred to 'invasion' here). Invasion was analyzed by infecting cells for 4–6 h at 35°C to allow the uptake of EB by the host cell (S1A Fig). At this time period, EB transform to RB and thus the bacteria do not replicate at this stage of the developmental cycle. After silencing total EphA2 expression by siRNA transfection (S1B Fig), *Ctr* invasion was reduced by 30% (S1C Fig). It was previously shown that PDI enzymatic activity is required for *Ctr* entry [26]. Silencing of PDI (S1B Fig) reduced the invasion rate of *Ctr* by 65% and by 87% if both EphA2 and PDI were silenced together (S1C Fig) confirming a role of both proteins in the uptake of EB. Blocking the ligand-binding domain of EphA2 with an antibody directed against the N-terminus of EphA2 reduced invasion of *Ctr* by 30% in a biopsy-derived human fallopian tube epithelial cells (Fimb) which are the primary target cells of chlamydial genital infections (S1D Fig), demonstrating that this is not a cell line specific effect. Thus, EphA2 is required for both efficient adherence as well as invasion of EB suggesting that it directly interacts with *Ctr* on the cell surface and this interaction is followed by the induced uptake of EB by the host cell.

Induced uptake of receptor tyrosine kinases frequently requires the kinase function of the receptor [28]. To get a first hint whether the kinase of EphA2 is required for *Ctr* entry, a clinically approved small molecule EphA2 tyrosine kinase inhibitor, dasatinib (DA), was used [29,30]. In comparison to the control treatment (DMSO), pretreatment of cells with 2.5 or 5 μM DA significantly reduced the entry of EB by 73% or 97% respectively (S1E Fig). Although we cannot exclude a role of other DA-sensitive kinases like Src family kinases, BCR-ABL, KIT (a cytokine receptor) and PDGFR [31,32], the drastic reduction in *Ctr* invasion demonstrates the functional relevance of RTKs for *Ctr* invasion.

### *Ctr* infection induces rapid EphA2 activation and quantitative receptor internalization

**Fig 2:**
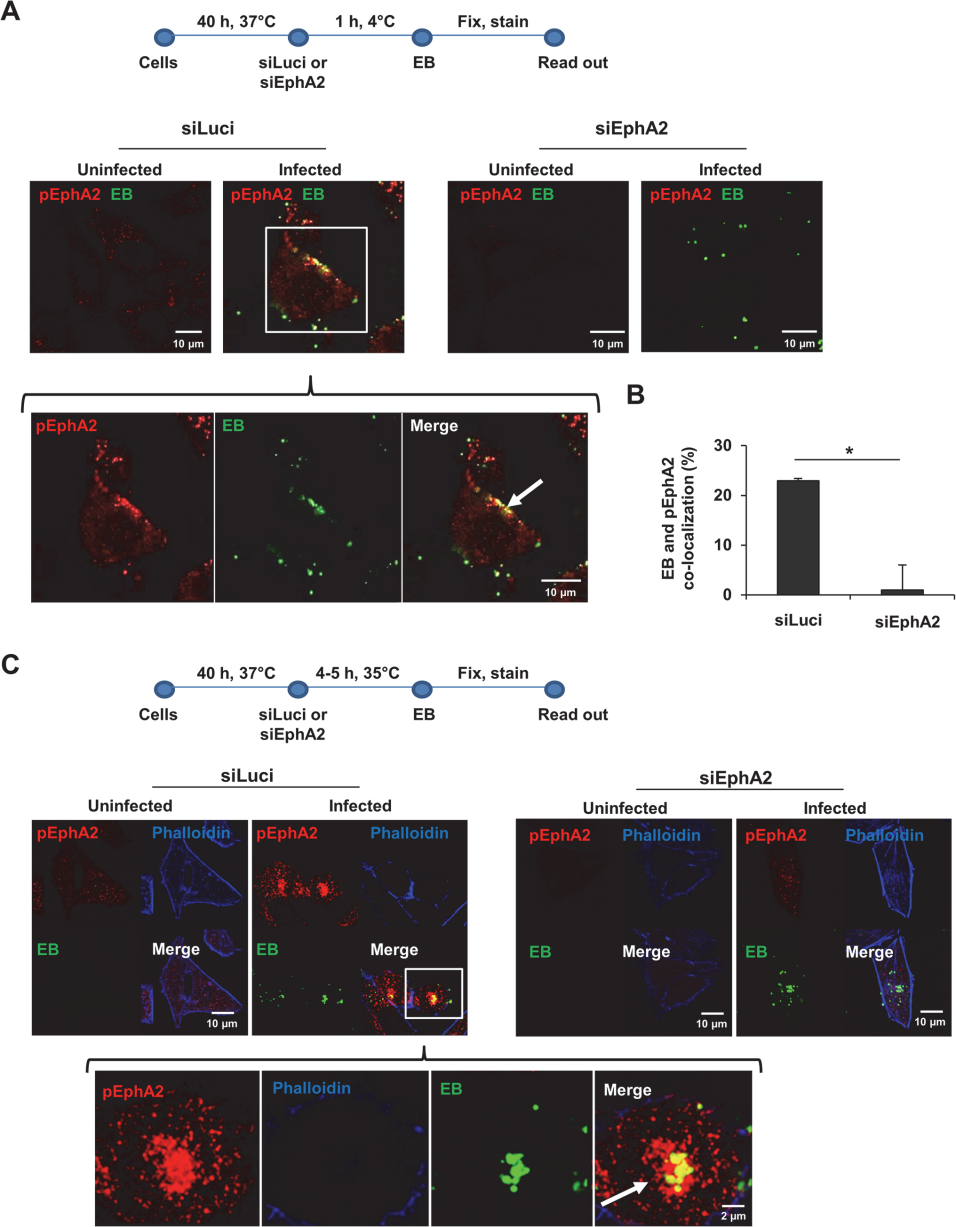
EphA2 activated upon early infection co-localizes with *Ctr*. (A) Adherence assay: HeLa cells were transfected with siRNA against luciferase gene (siLuci) or EphA2 gene (siEphA2) for 40 h at 37°C. The transfected cells were infected with *Ctr* (MOI-100) for 1 h at 4°C followed by immunostaining *Ctr*-EB (Hsp60, green) and pEphA2 (phospho EphA2 Ser897, red). (B) Co-localization of EB with pEphA2 was quantified. The graph was made similar to Fig 1B. The data are expressed as a mean percentage of pEphA2-associated EB (± SD) compared to total EB. *P<0.05. Error bars show mean ± SD. (C) Invasion assay: The transfected cells were infected with *Ctr* (MOI-20-25) for 4–5 h at 35°C followed by immunostaining as (A) including Actin filaments. (A, C) Arrows indicate the co-localization of *Ctr* with pEphA2 (yellow). Magnification is indicated in size bar.

Since surface EphA2 is required for *Ctr* adherence and invasion, we next investigated the activation of EphA2 upon *Ctr* infection. Phosphorylation of EphA2 at Ser897 (pEphA2), indicative of receptor activation, was detected close to adhering EB during the entry phase of chlamydial infection (Fig 2A), suggesting that interaction of EB induced receptor activation. As shown in Fig 2B, ~23% EB co-localized with pEphA2. Cross-reaction of the pEphA2-specific antibody could be excluded since no signal was detected upon knockdown of EphA2 expression (Fig 2A). We then asked where the activated EphA2 is located in the infected cell after receptor endocytosis. Interestingly, the internalized active EphA2 receptor not only localized directly to the invaded EB, but also accumulated close to the bacteria at 4–5 h post infection (p.i.) (Fig 2C). Thus, internalized EphA2 is stabilized and continues to signal after endocytosis in *Ctr*-infected cells.

We then overexpressed EphA2 to further substantiate its invasion supportive function and its association with invaded *Ctr*. The invasion rate of *Ctr* 6 h p.i. in EphA2 overexpressing cells was determined by FACS under permeabilised conditions. Increased EphA2 expression was confirmed in EphA2-pcDNA3-transfected cells compared to the control-transfected cells (S2A–S2C Fig). Invasion of *Ctr* increased about 2-fold if EphA2 was overexpressed (S2B and S2C Fig). Consistent with our previous observation (Fig 2C), invaded *Ctr* co-localized with EphA2 inside the cell (S2C Fig, indicated by arrows). These results confirm that EB bind and activate surface EphA2, resulting in *Ctr* and receptor internalization.

**Fig 3:**
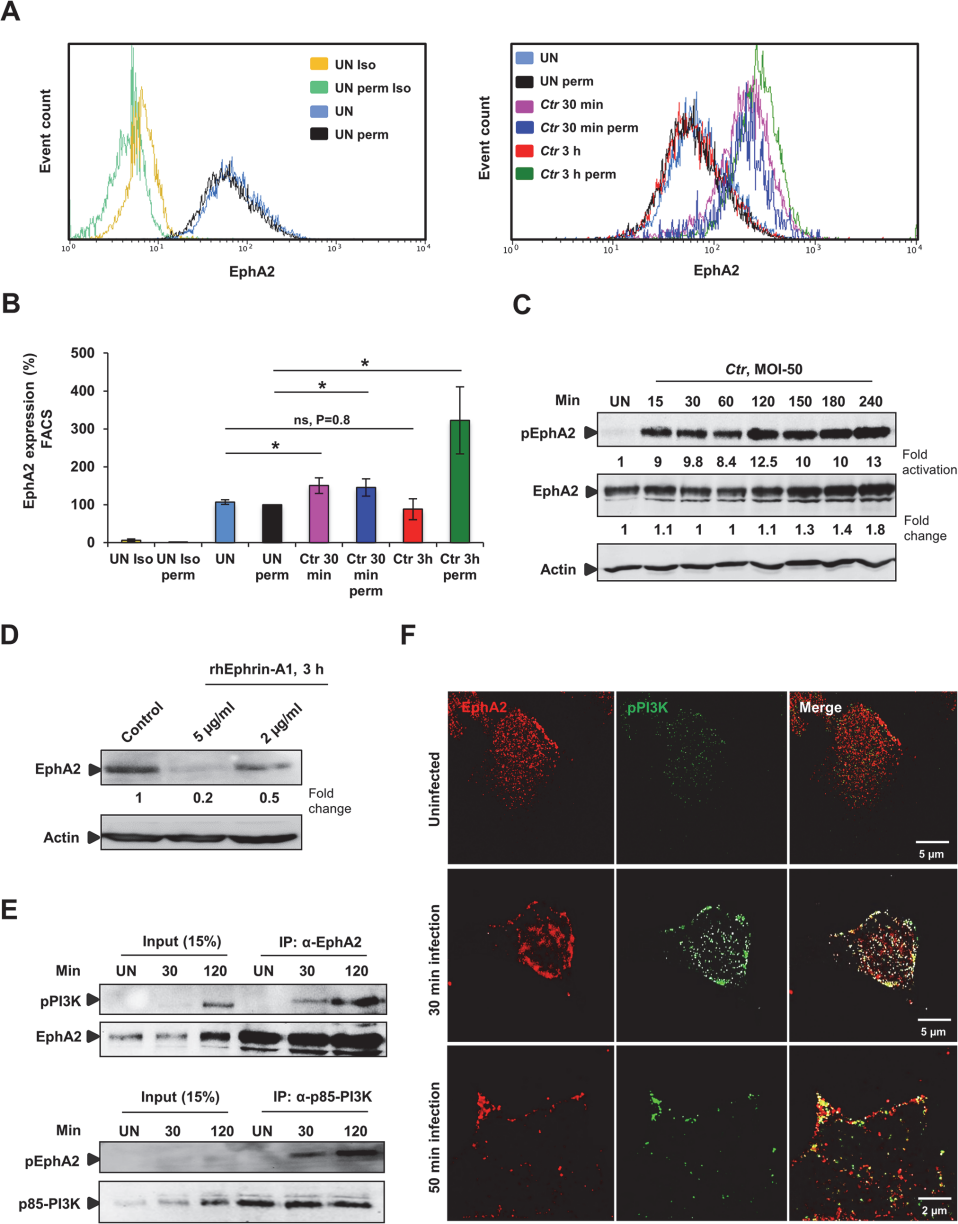
*Ctr*-induces EphA2 activation and receptor internalization which associates with pPI3K during early infection. (A) HeLa cells uninfected (UN) or infected with *Ctr* (MOI-50-75) for 30 min or 3 h were analyzed via FACS for surface EphA2 under non permeabilised or total EphA2 under permeabilised condition. Controls were indicated on the left curve chart and corresponding samples on the right curve chart of the same experiment. (UN: uninfected, Iso: isotype and perm: permeabilised). (B) Result of experiment shown in A demonstrated as bar chart. Shown is the mean ± SD of three independent experiments normalized to UN perm. *P<0.05, ns: non-significant. Error bars show mean ± SD. (C) HeLa cells were UN or infected with *Ctr* (MOI-50) for the indicated time points. The cells were immunobloted against pEphA2 and Actin. The blot was stripped and reprobed for total EphA2. (D) HeLa cells were treated with control or rhEphrin-A1 for 3 h and were harvested for WB analysis. (E) HeLa cells were UN or infected with EB for the indicated time points and immunoprecipitated (IP) with α-EphA2 or α-p85-PI3K antibodies. The IP material was solved in 40 μl Laemmli (100%) and loaded 20 μl for WB studies (50%) or (F) immunostained using α-EphA2 and α-pPI3K for 2 h at RT followed by secondary staining with anti-mouse Alexa fluor 488 and anti-rabbit Alexa fluor 647 for the microscopic analysis, respectively. Magnification is indicated in size bar.

Since our results so far demonstrated that the surface EphA2 is required for *Ctr* adherence and invasion, we next investigated whether surface display of EphA2 changes during *Ctr* entry. Interestingly, surface display of EphA2 increased as early as 30 min p.i., but returned to basal levels by 3 h p.i., as was determined by FACS under non-permeabilised condition measuring surface exposed EphA2 only (Fig 3A and 3B). This raised the question whether the rapid downregulation of surface EphA2 that follows the initial increase results from receptor internalization. FACS analysis under permeabilised conditions to detect surface exposed and internalized proteins showed an increased EphA2 signal at 3 h p.i., (Fig 3A and 3B). Since we could not detect EphA2 in the supernatant of cells at this time-point (S3A Fig), we concluded that EphA2 was internalized into the cell.

Internalization is probably mediated by binding of EB to the ligand binding domain of EphA2, since Ephrin-A1 ligand-induced aggregation of EphA2 on the cell surface has previously been shown to function as the internalization signal [33]. This ligand-induced activation of EphA2 results in auto-phosphorylation followed by rapid degradation. In line with receptor activation by *Ctr* infection, we observed an increased EphA2 phosphorylation, reaching a 9-fold increase as early as 15 min p.i. (Fig 3C). Activation of EphA2 depended on viable bacteria since heat-killed *Chlamydia* failed to induce EphA2 phosphorylation (S3B Fig). Interestingly, in contrast to ligand-induced EphA2 degradation ([33] and Fig 3D), EphA2 was not degraded in the cell during *Ctr* infection. Total levels of EphA2 rather started to increase around 4 h p.i., (Fig 3C), implying that EphA2 signaling induced by Ephrin-A1 and EB interaction differs. We, in addition, concluded that the rapid increase and decline in cell surface exposed EphA2 during early infection time-points is independent of changes in total EphA2.

### EphA2 activated by infection recruits and activates PI3 kinase

Infection with *Ctr* activates the Raf/MEK/ERK and PI3K/Akt pathways that support normal chlamydial development and keep the infected cell in an apoptosis resistant state [14]. The pathway that leads to PI3K activation has not been identified so far in *Ctr* infection. Several studies in recent years have shown the association of activated EphA2 with PI3K [34,35]. To reveal whether the activated EphA2 induced by *Ctr* infection interacts with PI3K, endogenous EphA2 or the endogenous p85 regulatory subunit of PI3K (p85-PI3K) was immunoprecipitated from the lysates of uninfected (UN) or *Ctr*-infected cells. Activated PI3K co-immunoprecipitated with EphA2 and activated EphA2 co-immunoprecipitated with p85-PI3K at 30 min p.i. and the activation of both proteins were increased when immunoprecipitated from cells infected for 2 h (Fig 3E). The interaction was further verified by microscopy showing the co-localization of EphA2 with activated PI3K after *Ctr* infection (Fig 3F). These results strongly suggest that EphA2 activated upon *Ctr* infection recruits and activates PI3K during early infection.

### Intracellular EphA2 associates with *Ctr* inclusion during mid-phase of the developmental cycle and is prevented from re-translocating to the cell surface

**Fig 4:**
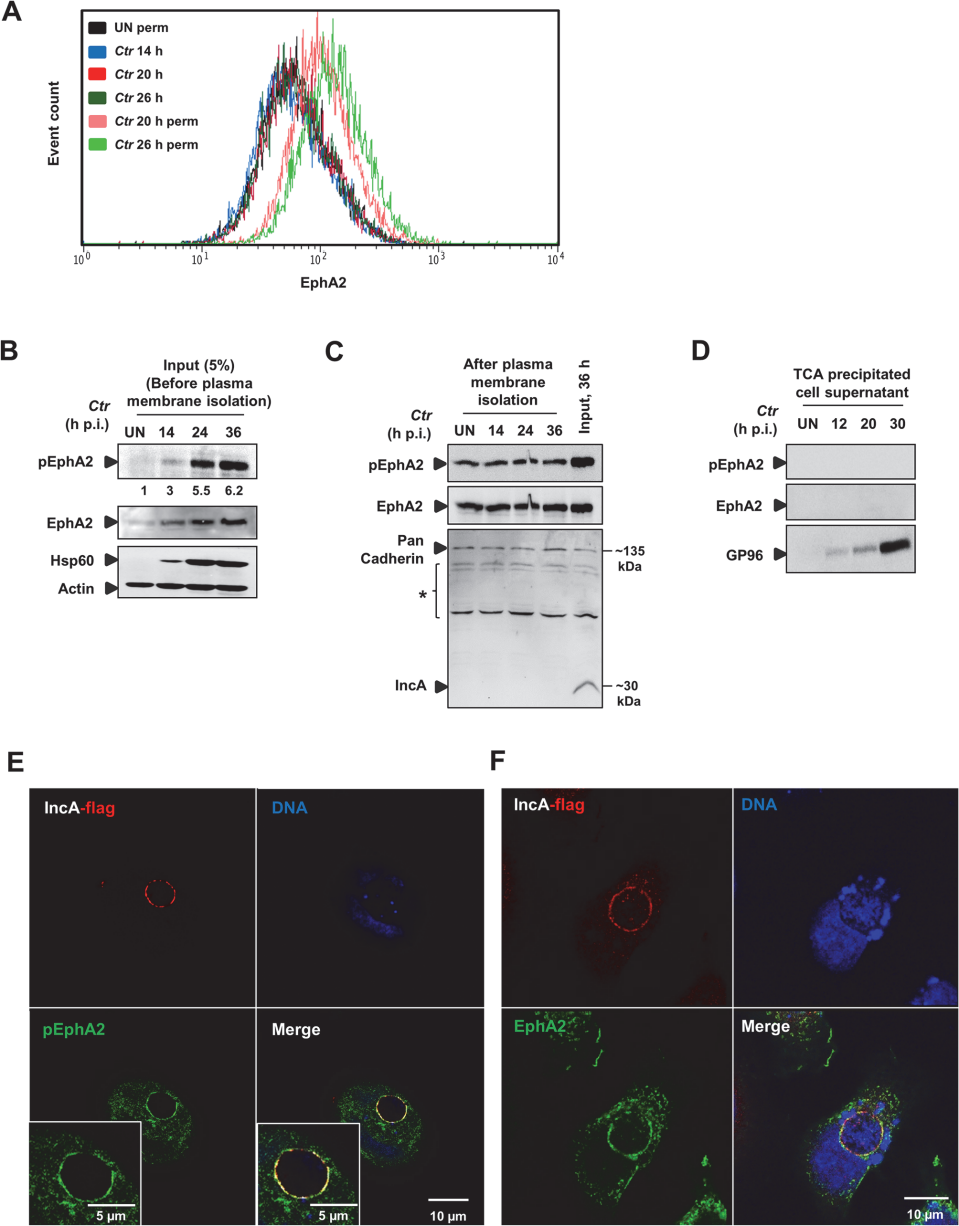
*Ctr*-induced EphA2 expression is prevented being re-translocated to the cell surface and is recruited to the inclusion membrane. (A) HeLa cells were infected with *Ctr-*EB (MOI-1) for 14, 20 and 26 h and cells were analyzed via FACS as Fig 3A. (B) HeLa cells were infected with *Ctr-*EB for 14, 24 and 36 h. Input (5%) was taken after homogenization step (before plasma membrane protein isolation) and subjected to WB analysis to determine pEphA2, Hsp60 and Actin. The blot was stripped and reprobed for total EphA2. Numbers under the blot represents fold activation for pEphA2 with respect to total EphA2. (C) Plasma membrane protein was isolated from UN or *Ctr*-infected cells at different time points and subjected to WB analysis. Pan Cadherin was used as a control for plasma membrane. IncA was used as a marker to test the quality of the isolated plasma membrane. * Unspecific bands detected by IncA polyclonal serum. (D) Culture medium of the UN as well as time course *Ctr-*infected cells (as indicated) was collected and TCA precipitated. The precipitated lysates were subjected to WB analysis against pEphA2, total EphA2 and GP96. (E, F) HeLa cells were transfected with EphA2-pcDNA3 at 37°C and the cells were infected with *Ctr*-pIncA-flag for 24 h at 35°C. (E) Cells were fixed and stained against Flag (red), EphA2 (green) and DNA (blue). (F) Cells were fixed and stained against Flag (red), pEphA2 (green) and DNA (blue). Magnification is indicated in size bar.

To this point, all data on the role of EphA2 during chlamydial infection were obtained during the early phase of infection and prior to EB-RB transformation. We next determined the levels of EphA2 on the cell surface as well as inside the cell. Surface EphA2 expression was similar at three different time points of *Ctr* infection (14, 20 and 26 h p.i.) whereas permeabilised-infected cells (20 and 26 h p.i.) displayed a clear increase of intracellular EphA2 levels (Fig 4A). Consistently, the total and activated levels of EphA2 increased with increasing infection times (14, 20 and 36 h) as was detected by immunoblot analysis of cells infected with viable (Fig 4B), but not heat-killed bacteria (S3B Fig). The amount and phosphorylation of EphA2 increased during *Ctr* infection in HeLa, HUVEC and also in Fimb cells (S3C and S3D Fig), demonstrating that this is not a cell line specific effect. The increased transcriptional levels of EphA2 upon *Ctr* infection in HeLa cells according to [36], correlates to the increased translational levels of EphA2 (S3C Fig). Further, we also tested the expression of PDGFRβ that has been shown to be a receptor for *C*. *muridarum* [8] and of PDI required for *Ctr* invasion [26] as well as of EphB4, which is not required for *Ctr* adherence (Fig 1G). In contrast to EphA2, PDGFRβ is downregulated during *Ctr* infection and no significant changes were observed for PDI or EphB4 expression (S3E Fig), suggesting that EphA2 is specifically upregulated and activated during *Ctr* infection.

We then investigated where the increased and activated EphA2 is located in the infected cell during the mid-phase of *Ctr* infection. Plasma membrane proteins isolated from the infected cells at those time points contained constant levels of total and activated EphA2, suggesting that the basal levels of EphA2 and activated EphA2 at these time points were unchanged at the cell surface (Fig 4C). Further, we also precipitated the supernatants of infected cells to test if surface EphA2 is shed into the culture medium during infection as previously demonstrated for GP96 (Karunakaran et al., submitted). EphA2 was not detectable in the culture supernatant (Fig 4D), confirming that the increase in levels of EphA2 and activated EphA2 is restricted to the intracellular space.

To further define the intracellular localization of EphA2, cells were first transfected with EphA2-pcDNA3 and infected for different time intervals with *Ctr*. Overexpressed EphA2 was detected in the vicinity (6 and 16 h p.i.) and in association (24 and 40 h p.i.) with the inclusion (S4A and S4B Fig). To obtain further proof for the localization and activation of EphA2 at and around the inclusion membrane, the inclusion membrane-associated chlamydial IncA protein was fused to a HA and Flag tag in the plasmid pGFP::SW2 [23], replacing the GFP:CAT (S4C Fig). The *Ctr* strain expressing recombinant IncA-HA-Flag (*Ctr*-pIncA-flag) was confirmed by WB analysis (S4D Fig). In cells infected with *Ctr*-pIncA-flag, EphA2 remained active and co-localized to the chlamydial inclusion membrane (Fig 4E and 4F), demonstrating that the inclusion-associated EphA2 receptor is active.

### EphA2 is required for long-lasting PI3K activation

**Fig 5:**
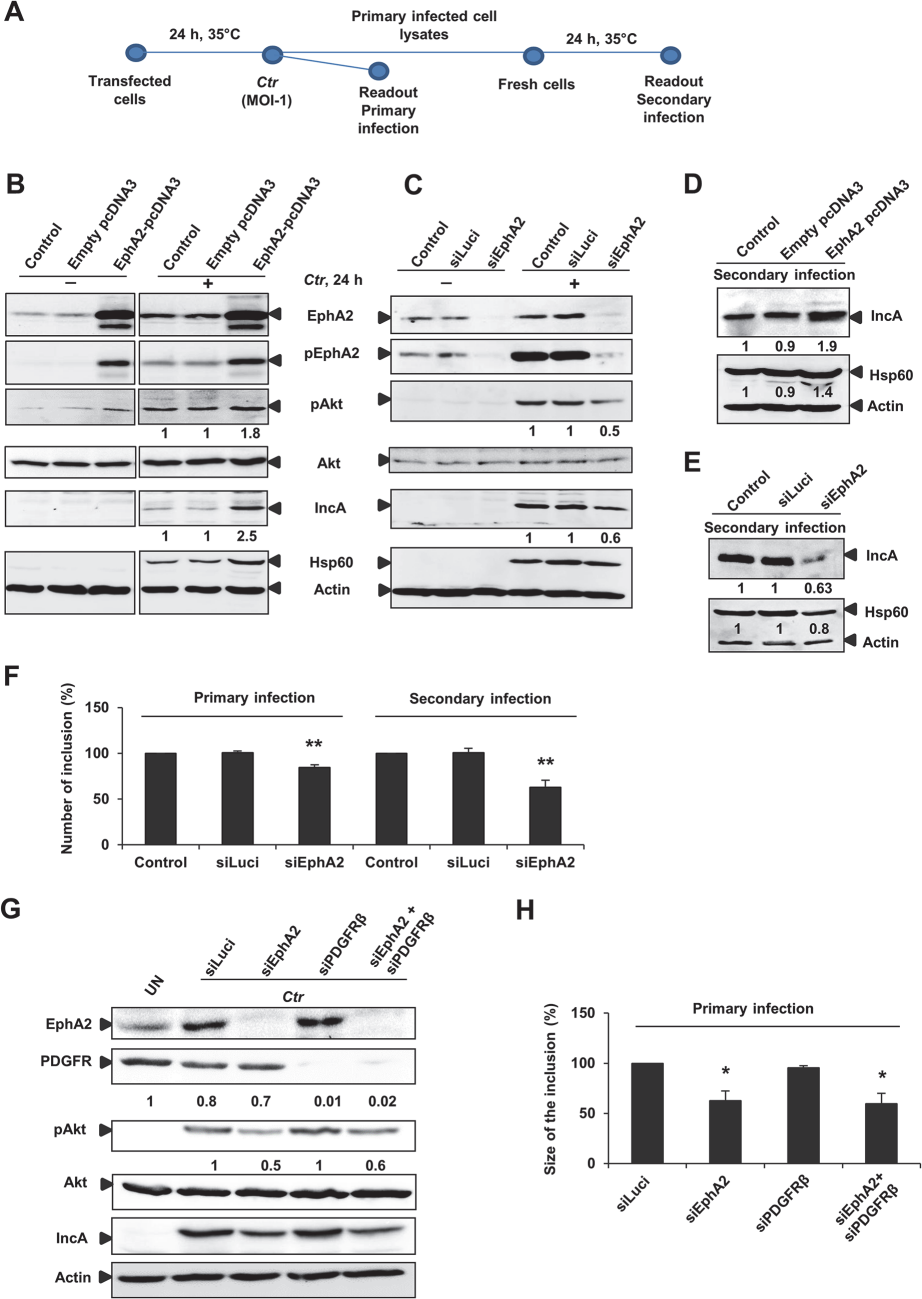
EphA2 overexpression and knockdown influences *Ctr* infection. (A) Infectivity assay performed for B) C) D) E) F): The transfected cells infected with *Ctr* (MOI-1) for 24 h at 35°C was referred as primary infection. The supernatant of the primary infected cells was taken to infect the fresh cells to determine the secondary infection (see infectivity assay-methods). (B) HeLa cells were left untransfected (control) or transfected with empty pcDNA3 or EphA2-pcDNA3 each 1 μg/ml and then left UN or infected with *Ctr* for 24 h. Cells were harvested for WB analysis. (C) HeLa cells were untransfected (control) or transfected with siRNA against luciferase gene (siLuci) or EphA2 gene (siEphA2) for 40 h at 37°C and then left UN or infected with *Ctr* for another 24 h. Cells were harvested for WB analysis. (D, E) Primary infected cell lysates of (B) and (C) were taken to infect the fresh cells for secondary infection (Infectivity assay). (F) Number of the inclusion per cell (%) for the infectivity assay was determined by counting the inclusion on 10 independent fields. Shown is the mean ± SD of three independent experiments normalized to control. **P<0.01. Error bars show mean ± SD. (G) Cells were transfected with siRNA against luciferase (siLuci) or EphA2 (siEphA2) or PDGFRβ (siPDGFRβ) or both together for 40 h at 37°C and then infected with *Ctr*. UN: uninfected cells. Cells were harvested to determine the respective proteins by WB analysis. (H) Size of the inclusion per cell (%) for (F) was determined by ImageJ software by measuring cells out of four microscopic fields. Shown is the mean ± SD of two independent experiments normalized to siLuci. *P<0.05. Error bars show mean ± SD. In all of the above experiments, numbers under the blot represents fold activation for phospho specific proteins with respect to total proteins and fold change for the total proteins.

The rapid and strong activation of EphA2 by chlamydial infection and the association of EphA2 with inclusion during the replicative phase of the developmental cycle suggested that EphA2 is required for chlamydial development, probably by signaling via PI3K. It is important to note that experiments conducted to investigate the role of EphA2 during the mid-phase of the chlamydial cycle were performed with a low MOI of 1–2 (in contrast to MOI of 50–100 for adherence and MOI of 20–50 for invasion experiments) to ensure similar initial infection irrespective of the EphA2 status (Infectivity assay-Fig 5A). Indeed, EphA2 overexpression resulted in increased pAkt levels in uninfected cells, which further increased upon *Ctr* infection (Fig 5B). In line with a role of EphA2 in PI3K activation also at the mid-phase of the chlamydial cycle, Akt activation was reduced upon knockdown of EphA2 in *Ctr*-infected cells (Fig 5C).

Interfering with pAkt signaling by modulating EphA2 levels also influenced *Ctr* primary and progeny infection (Fig 5B and 5H). EphA2 overexpression or EphA2 silencing changed the progeny infection by approximately 48% (increase during overexpression) and 37% (decrease during knockdown), respectively (Fig 5D and 5E). Number of inclusion in both primary (18% reduction) and secondary infection (43% reduction, Fig 5F) as well as the size of the inclusion in primary infection (40% reduction, Fig 5H) were significantly affected upon silencing EphA2 expression. Further, silencing of PDGFRβ had no effect on PI3K activation and *Ctr* infection (Fig 5G and 5H), demonstrating a specific role of EphA2 in *Ctr* infection. These results suggested that EphA2 activates Akt and thereby supports chlamydial development.

**Fig 6:**
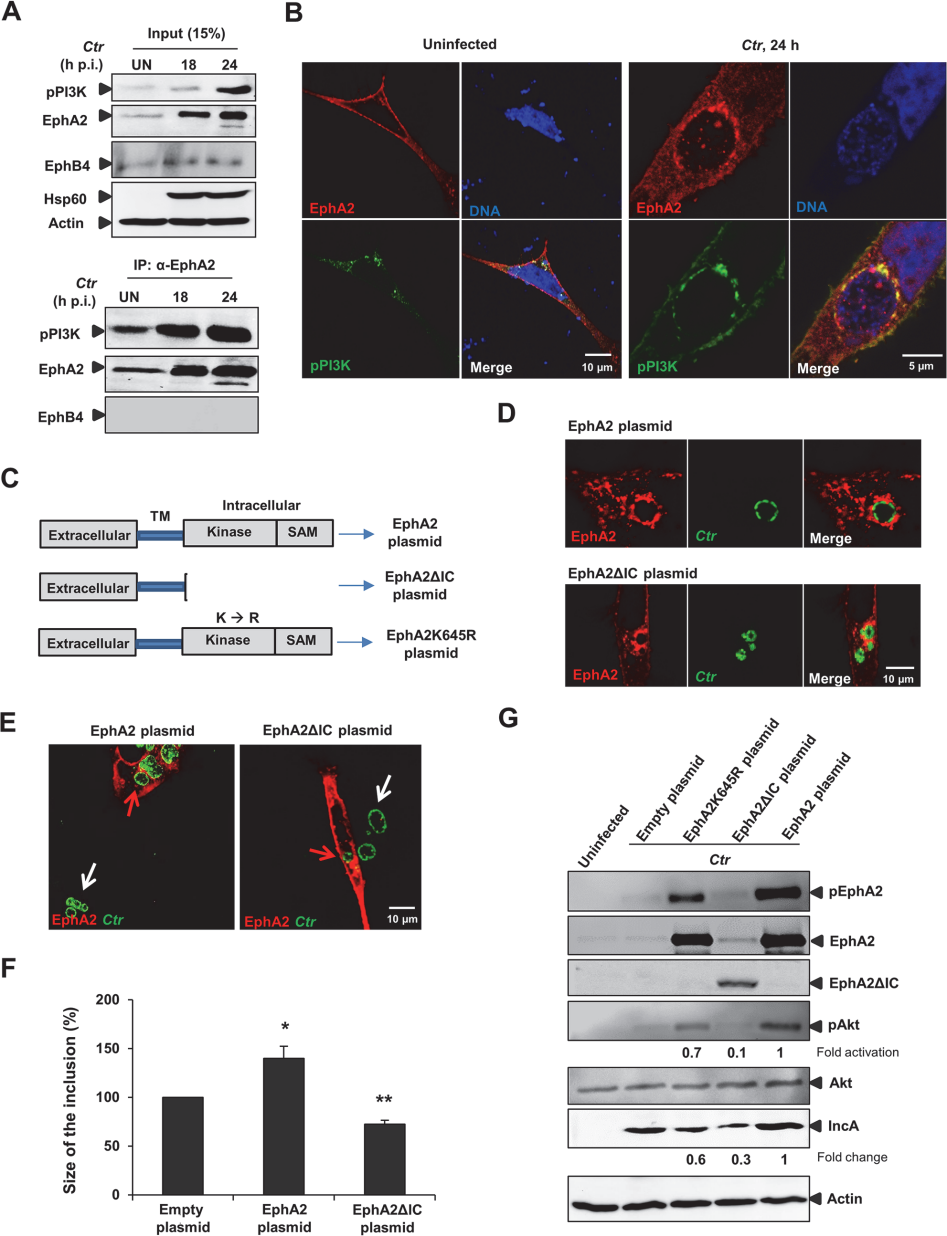
EphA2 intracellular cytoplasmic domain is crucial for *Ctr* infection. (A) HeLa cells were left UN or infected with *Ctr* (MOI-2) for the indicated period of times and immunoprecipitated using α-EphA2 antibody. The IP material was solved in 40 μl Laemmli (100%) and loaded 20 μl for WB studies (50%). (B) Cells were transfected with EphA2-pcDNA3 and p85-PI3K expression plasmid together for 15 h followed by *Ctr* infection for 24 h. Cells were stained against EphA2 (red), DNA (blue) and pPI3K (green). (C) Schematic representation of full length EphA2 plasmid, EphA2 kinase dead mutant plasmid (EphA2K645R) and EphA2 mutant plasmid missing the entire cytoplasmic domain (EphA2ΔIC). TM: transmembrane domain. (D) The plasmids having full length EphA2 or EphA2ΔIC were transfected and these cells were infected with *Ctr* for 20 h. Cells were fixed and stained against α-EphA2 antibody (red) and *Ctr* using α-Hsp60 (green). (E) Arrows were marked to illustrate the difference between the size of the inclusion of untransfected (white arrows) and transfected (red arrows) cells. (F) Size of the inclusion for (E) was determined by ImageJ software. Empty-plasmid transfected cells act as a control. Shown is the mean ± SD of two independent experiments normalized to empty-plasmid transfected cells. *P<0.05, **P<0.01. Error bars show mean ± SD. (G) Cells after transfection of the constructs (each 1.5 μg/ml) indicated above the lanes followed by infection (MOI-1) for 18 h were subjected to WB analysis to analyze the proteins as indicated. EphA2ΔIC was detected by N-terminal EphA2 antibody. Due to the high intensity of total EphA2 and phospho EphA2 from the EphA2-transfected infected cells, the upregulation of endogenous EphA2 cannot be visualized. (B, D, F) Magnification is indicated in size bar.

As we found increased Akt activation in EphA2 overexpressing-infected cells (Fig 5B) and interaction of activated PI3K with EphA2 during early infection (Fig 3E and 3F), we next determined whether increased total EphA2 during late *Ctr* infection also associates with activated PI3K. Co-immunoprecipitation experiment confirmed the presence of increased endogenous levels of activated PI3K with the immunoprecipitated endogenous EphA2 at 18 and 24 h p.i, when *Ctr* has reached the replicative state (Fig 6A). The interaction was further verified by confocal microscopy showing the co-localization of EphA2 with activated PI3K after *Ctr* infection (Fig 6B), suggesting that PI3K activation is an important function of intracellular EphA2 in *Ctr* infection.

### EphA2 intracellular cytoplasmic domain function is essential for chlamydial development

We next asked whether the intracellular domain function of EphA2 is required for supporting *Ctr* development and PI3K activation. Therefore, an EphA2 deletion mutant lacking the cytoplasmic domain including the kinase domain and SAM domain (EphA2ΔIC) was constructed (Fig 6C). EphA2ΔIC localized to inclusion like the wildtype form and was expected to function in a dominant-negative way by competing with the endogenous EphA2 receptors for binding to EB without activating downstream signaling pathways (Fig 6D). Transient overexpression of either EphA2 or EphA2ΔIC had a profound effect on the inclusion size. In cells transfected with full length EphA2, the size of the inclusion appeared bigger compared to the untransfected neighboring cells (Fig 6E and 6F, see also S4B and S5A and S5B Fig). In contrast, the inclusion size appeared smaller in EphA2ΔIC transfected cells compared to untransfected cells (Fig 6E and 6F). Interestingly, Akt activation was reduced in EphA2ΔIC transfected cells (Fig 6G) suggesting that EphA2ΔIC interferes with signaling of endogenous EphA2 in a dominant negative manner, e.g. by preventing functional dimer formation. These results clearly demonstrate the importance of EphA2 intracellular cytoplasmic domain function for *Ctr* infection.

**Fig 7:**
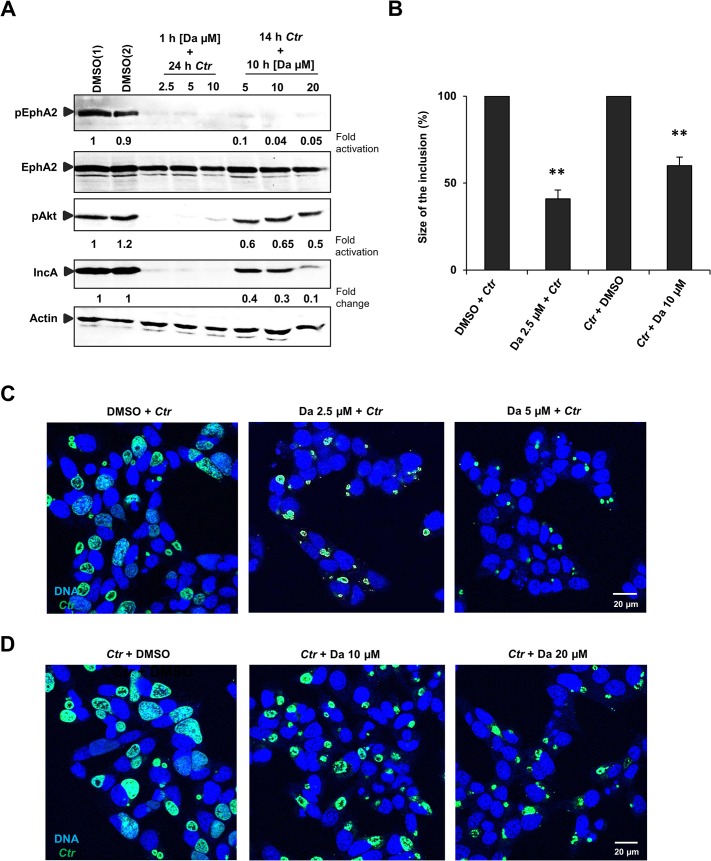
Small molecule EphA2 inhibitor dasatinib affects *Ctr* infection. (A) HUVEC cells were pretreated with DMSO(1) control or with 2.5 μM, 5 μM and 10 μM of DA, respectively, for 1 h at 37°C and were infected with *Ctr* (MOI-1) for 24 h. Or the cells were first infected with *Ctr* for 14 h and then treated with DMSO(2) control or with 5 μM, 10 μM and 20 μM DA, respectively, for 10 h. Cells were harvested for WB analysis. (B, C, D) Experiments were performed as (A) and immunostained for *Ctr* inclusion (Hsp60, green) and DNA (Draq5, blue). (B) The size of the inclusion was measured by ImageJ software. Shown is the mean ± SD of two independent experiments normalized to control. **P<0.01. Error bars show mean ± SD. Magnification is indicated in size bar.

We then made use of the RTK inhibitor dasatinib (DA) to further investigate the role of the kinase activity of endogenous EphA2 for *Ctr* infection. Pretreatment of cells with DA for 1 h followed by 24 h of infection inhibited EphA2 activation (Fig 7A) and strongly affected *Ctr* infection (Fig 7B and 7C). DA may also inhibit Src family kinases, BCR-ABL, other RTKs like PDGFRβ, which has previously been shown to be required for *C*. *muridarum* entry [8]. However, PDGFRβ is rather downregulated during *Ctr* infection and additional silencing of PDGFRβ had no effect on *Ctr*-induced PI3K activation (Fig 5G) or inclusion size (Fig 5H). Also, co-silencing EphA2 together with PDGFRβ had no additive effect (Fig 5H), suggesting that *Ctr* does not employ the PDGFRβ during mid-phase infection.

To further substantiate the role of EphA2 kinase activity for chlamydial replication and development, cells were infected with *Ctr* for 14 h followed by 10 h treatment with DA. These conditions allowed normal inclusion formation and EB to RB transition prior to the inhibition of EphA2 kinase during the replicative phase of *Ctr* development. This strategy did not only inhibit the *Ctr*-induced EphA2 activation (Fig 7A) but also reduced the *Ctr* infection in a dose-dependent manner (Fig 7A and 7D). Akt phosphorylation was only mildly affected when DA was added at 14 h p.i., despite a reduction in *Ctr* infection (Fig 7A).

Since DA is known to block also other tyrosine kinases, we verified the importance of the EphA2 kinase activity for *Ctr* infection by a dominant negative approach. We, therefore, generated an EphA2 kinase dead mutant (EphA2K645R) (Fig 6C) as demonstrated previously [37]. The levels of activated Akt and *Ctr* infection were clearly affected in cells expressing EphA2K645R compared to full length EphA2. However, the effect was not as prominent as in cells expressing EphA2ΔIC (entire cytoplasmic deletion) (Fig 6G) suggesting that the EphA2 cytoplasmic domain besides the kinase activity has additional functions for *Ctr* development.

### *Ctr*-induced EphA2 upregulation is mediated by ERK signaling

**Fig 8:**
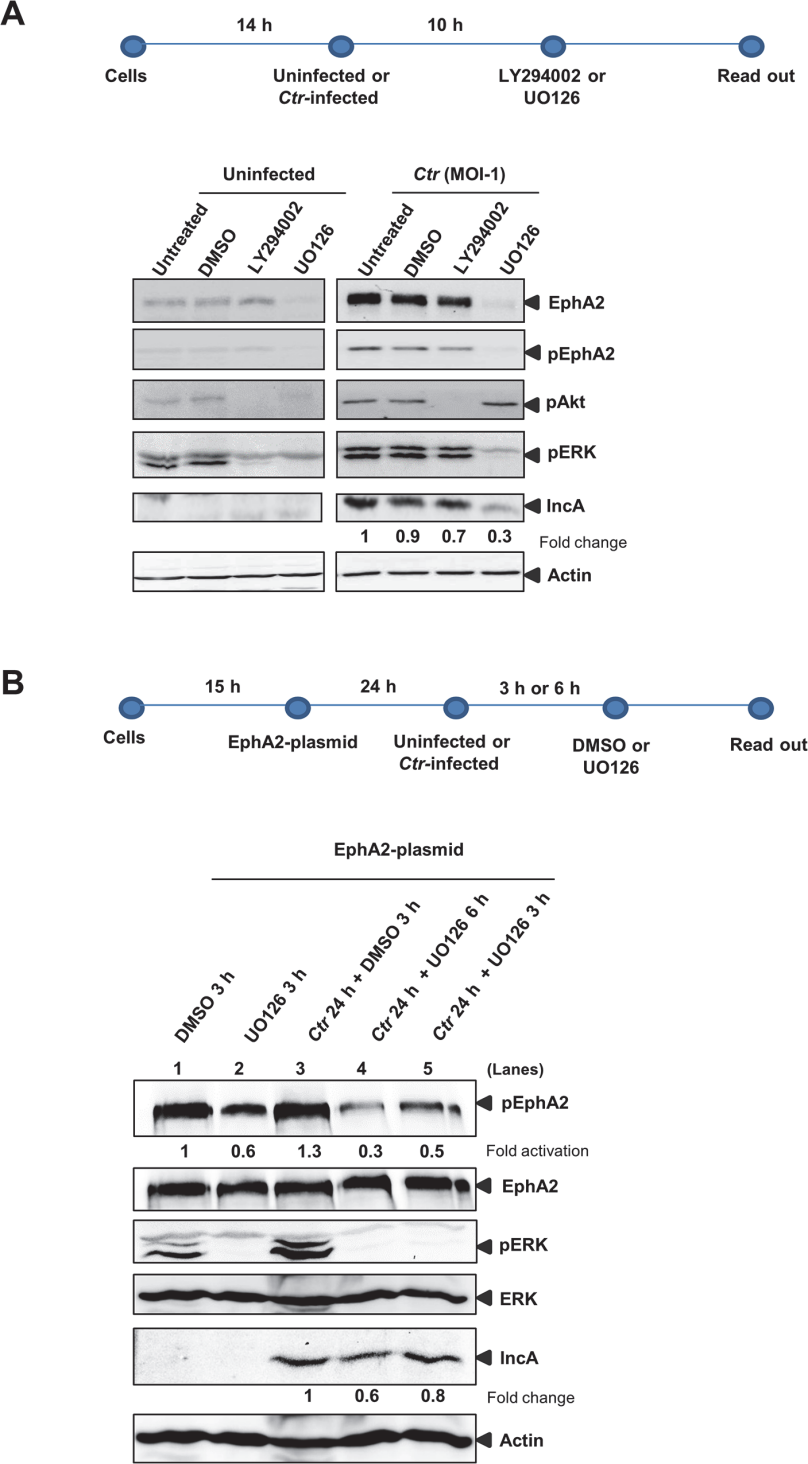
*Ctr*-mediated EphA2 regulation is ERK dependent. (A) HUVEC cells were left uninfected or infected with *Ctr* (MOI-1) for 14 h followed by treatment with DMSO or PI3K inhibitor LY294002 (15 μM) or MAPK inhibitor UO126 (15 μM) for 10 h. After 24 h of total infection, cells were subjected to WB analysis against respective proteins. (B) EphA2 plasmid-transfected HeLa cells were left uninfected or infected with *Ctr* for 24 h as mentioned above the lanes. The cells were treated with DMSO or UO126 (30 μM) for 3 h or 6 h as described above the lanes and subjected to WB analysis to determine the respective proteins as indicated.

The strong and long-lasting upregulation of EphA2 expression leading to its accumulation around the inclusion implied that infection-induced signaling pathways are involved. We, therefore, investigated the role of the MAPK (Ras/Raf/MEK/ERK) and PI3K pathways in upregulation of EphA2 during *Ctr* infection. Cells were left uninfected or infected for 14 h followed by treatment with MEK-1 inhibitor U0126 or the PI3K inhibitor LY294002 to allow inclusion formation prior to application of chemical inhibitors. LY294002 treatment after 14 h of *Ctr* infection did not affect total EphA2 levels or its activation whereas treatment with U0126 strongly reduced total and activated EphA2 levels (Fig 8A) and *Ctr* infection (see IncA in Fig 8A).

To discriminate if *Ctr*-mediated ERK activation is also essential for EphA2 activation (and not only for increased expression), EphA2 was overexpressed under the control of CMV promoter (pcDNA3 plasmid) and the effect of ERK inhibition before and after 24 h p.i., was investigated (Fig 8B). EphA2 overexpressed by transfection of an expression construct was not affected by ERK inhibition in both uninfected and *Ctr*-infected cells (Fig 8B). Interestingly, after inhibition of ERK by UO126, the level of activated EphA2 was affected in both EphA2 transfected uninfected as well as in *Ctr*-infected cells (lanes 2, 4 and 5 in Fig 8B). These data suggest that infection-mediated ERK activation is necessary for increased EphA2 expression and also for the activation of EphA2 during infection.

### EphA2 is required to inhibit apoptosis signaling in infected cells

**Fig 9:**
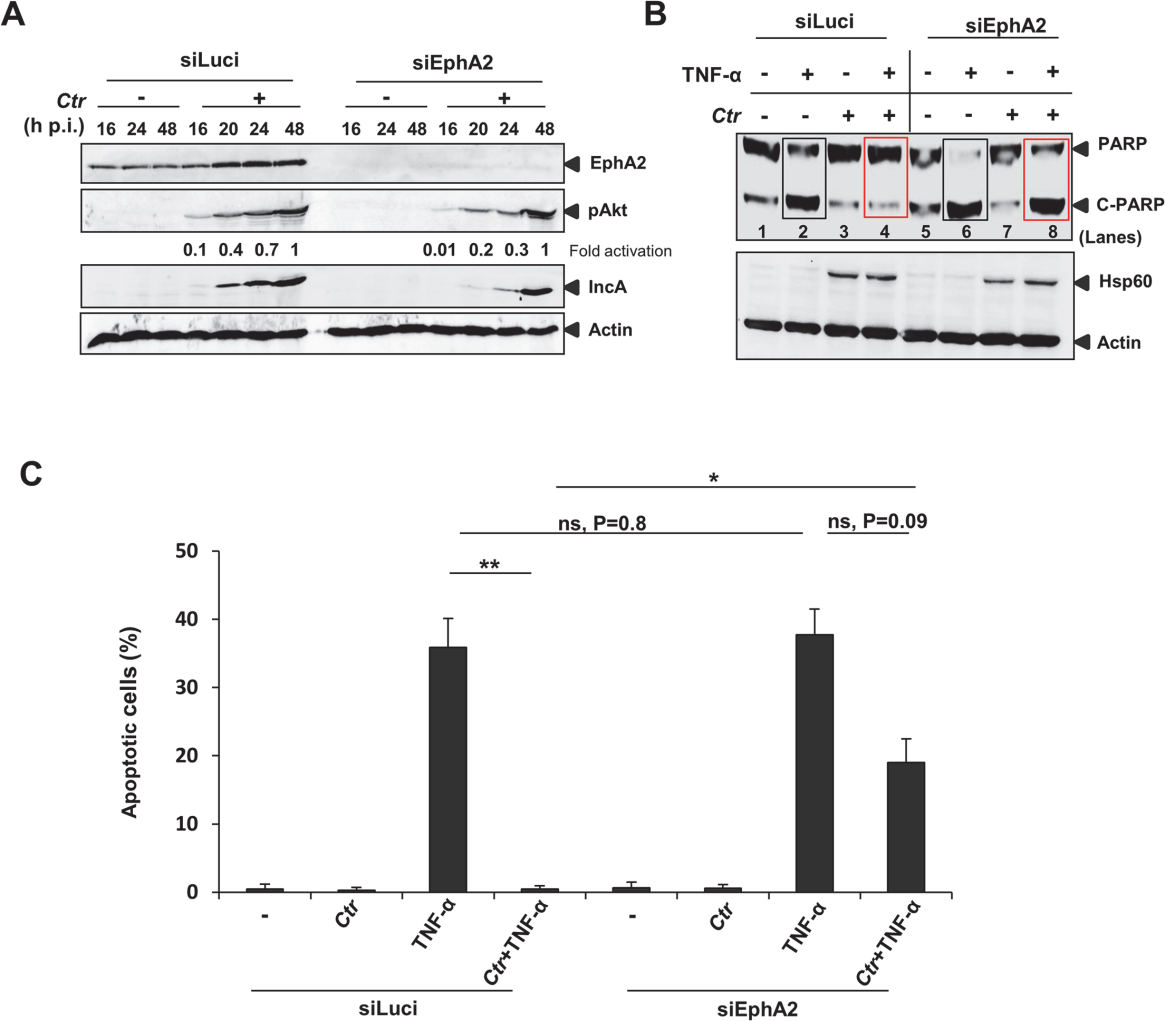
*Ctr*-infected cells were sensitized to TNF-α induced apoptosis upon EphA2 knockdown. (A) The transfection efficiency of siRNA directed against EphA2 was monitored by WB analysis against total EphA2. In addition, levels of pAkt, IncA and Actin were verified. (B) siLuci or siEphA2-transfected cells were left uninfected or infected with *Ctr* for 16 h. Cells were induced to apoptosis by TNF-α (50 ng/ml)/CHX (5 μg/ml) for 5–6 h. Processing of PARP, Hsp60 and Actin were monitored by WB analysis. Rectangle boxes (black: before infection) or (red: after infection) denotes the difference in PARP cleavage after TNF-α induction in siLuci and siEphA2 transfected cells. (C) For quantification, TUNEL positive cells from each sample were counted from ten different fields. Shown is the mean ± SD of two independent experiments. **P<0.01, *P<0.05, ns: non-significant. Error bars show mean ± SD.

*Chlamydia* infection is known to interfere with apoptosis signaling induced by various stimuli like staurosporine, etoposide, TNF-α, FAS antibody and granzyme B/perforin [14,38]. Since EphA2 plays a role in anti-apoptosis signaling [39,40] and the ERK pathway is required for *Ctr*-induced apoptosis inhibition [14] and EphA2 activation (Fig 8A and 8B), we tested the role of EphA2 in apoptosis inhibition of infected cells. Akt activation is dependent on EphA2 during mid-phase (16 to 24 h) but not late-phase *Ctr* infection (48 h) (Fig 9A), in line with its role in anti-apoptosis signaling during the replicative phase. Consistently, silencing of EphA2 sensitized cells that had been infected for 16 h to apoptosis induced by TNF-α/CHX determined by PARP cleavage and apoptotic cell count (Fig 9B and 9C). Thus, EphA2-mediated signaling also supports apoptosis resistance of *Ctr*-infected cells, demonstrating its crucial intracellular role in *Ctr* infection.

### *C. trachomatis*-serovar D utilizes EphA2 for entry and to prevent apoptosis

**Fig 10:**
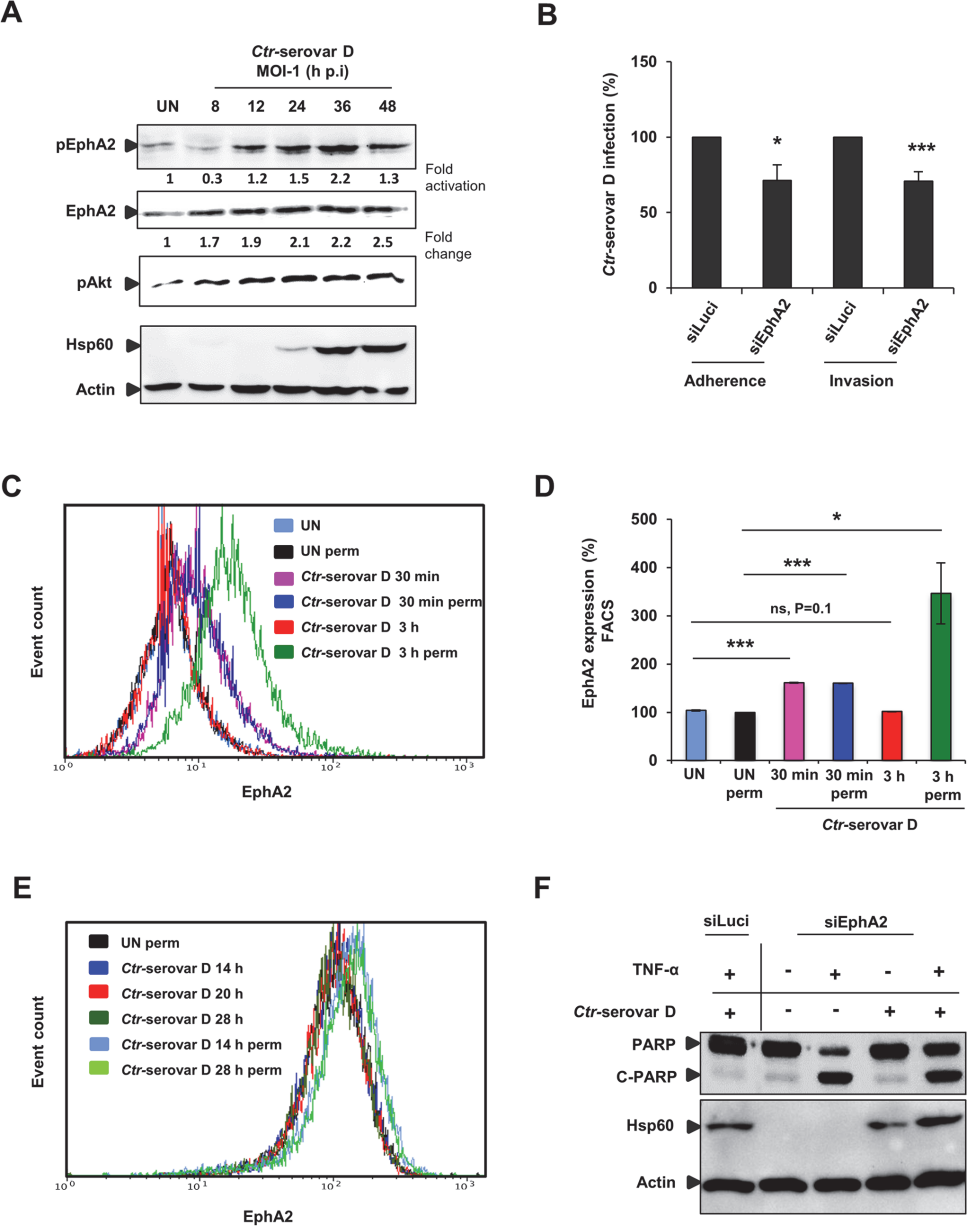
*Ctr*-serovar D utilize EphA2 signaling to invade the cells and to prevent apoptosis induced by TNF-α. (A) HeLa cells left UN or infected with *Ctr*-serovar D were centrifuged at 910 x g for 30 min and allowed to infect for the indicated time points. The cells were harvested and subjected to WB analysis. (B) HeLa cells were transfected with siRNA against luciferase gene (siLuci) or EphA2 gene (siEphA2) for 40 h at 37°C. Adherence assay: The transfected cells were infected with *Ctr-*serovar D for 1 h at 4°C. Cells were immunostained against EB and Actin filaments. Number of extracellular EB was counted randomly from 30 different cells. Data are expressed as percentage of extracellular EB relative to siLuci. Shown is the mean ± SD of three independent experiments normalized to siLuci. *P<0.05. Error bars show mean ± SD. Invasion assay: The transfected cells were infected with *Ctr-*serovar D for 4–5 h at 35°C. Cells were immunostained against EB and Actin filaments. Number of invaded EB was counted randomly from 30 different cells. Data are expressed as percentage of invaded EB relative to siLuci. Shown is the mean ± SD of three independent experiments normalized to siLuci. *P<0.001. Error bars show mean ± SD. (C) FACS analysis was performed with *Ctr*-serovar D infected cells as Fig 3A. (UN: uninfected, Iso: isotype and perm: permeabilised). (D) Results of C were shown as bar charts. Shown is the mean ± SD of two independent experiments normalized to UN. ***P<0.001, *P<0.05, ns: non-significant. Error bars show mean ± SD. (E) HeLa cells were left UN or infected with *Ctr-*serovar D (MOI-1) for 14, 20 and 28 h and cells were analyzed via FACS as Fig 4A. (F) EphA2 knockdown followed by 16 h *Ctr*-serovar D infected cells were induced to apoptosis by TNF-α (50 ng/ml)/CHX (5 μg/ml) for 5–6 h. Processing of PARP, Hsp60 and Actin were monitored by WB analysis.

We next investigated and confirmed that EphA2 is also upregulated and activated in cells infected with *C*. *pneumoniae* and other *Ctr* serovars like the ocular serovar A and the urogenital serovar D (S6A and S6B Fig and Fig 10A). We further tested if EphA2 is required for efficient binding and invasion of *Ctr*-serovar D to HeLa cells like observed for serovar L2. After silencing EphA2 expression, *Ctr-*serovar D binding and invasion were reduced by 28% and 30% respectively (Fig 10B). Interestingly, like previously observed for serovar L2, FACS analysis under non-permeabilised conditions revealed an increased expression of surface EphA2 as early as 30 min p.i., which returned to basal levels by 3 h p.i. (Fig 10C and 10D). The amount of total EphA2 in contrast was strongly increased at 3 h p.i. as was demonstrated by FACS analysis under permeabilised conditions (Fig 10C and 10D). Thus, we conclude that *Ctr*-serovar D also utilizes EphA2 to invade host cells. We next also determined the surface and total levels of EphA2 during the mid-phase infection with *Ctr*-serovar D. Like for serovar L2, surface EphA2 expression of cells infected with *Ctr*-serovar D was similar at three different time points of infection (14, 20 and 28 h p.i.) whereas permeabilised-infected cells displayed an increase of intracellular EphA2 levels (Fig 10E). We then also investigated the role of internalized EphA2 for apoptosis resistance induced by infection with *Ctr*-serovar D. EphA2 silenced cells upon 16 h p.i. were sensitized for TNF-α/CHX-induced apoptosis determined by PARP cleavage (Fig 10F). Thus, EphA2-mediated signaling is essential for invasion and apoptosis resistance of *Ctr-*serovar D infection.

## Discussion

First identified in a cancer cell line called erythropoietin-producing hepatocellular [41], numerous Eph receptors were subsequently detected, exhibiting diverse functions in vascular development, cancer and virus infection. Here, we report on the identification of EphA2 as a key factor of *Ctr* entry and development using an unbiased approach, based on the compilation of data from hypothesis-free proteomics and RNA interference approaches.

**Fig 11:**
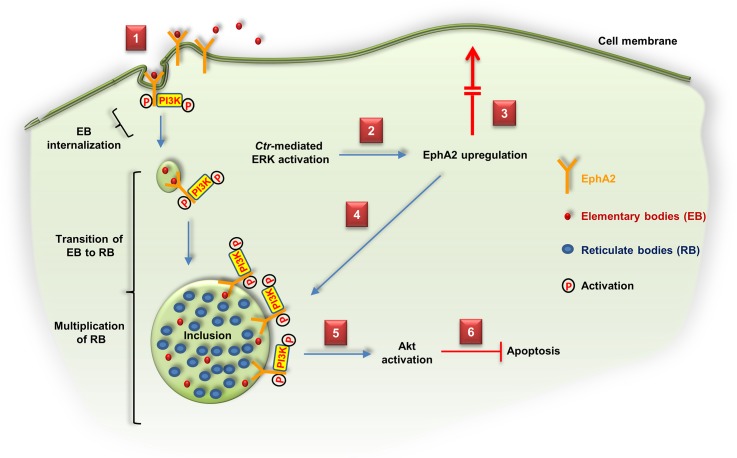
Schematic model depicting the role of EphA2 as an invasion and intracellular signaling receptor for *C*. *trachomatis*. (1) *Ctr*-EB bind and activate EphA2 and enter the cell together with the activated receptor and PI3K recruited to EphA2. (2) *Ctr*-mediated ERK activation induces EphA2 upregulation. (3) Upregulated EphA2 does not appear at the cell surface. (4) Intracellular EphA2 is associated with the *Ctr* inclusion which interacts with pPI3K. (5, 6) EphA2-induced signaling cascades (pAkt) are required to inhibit apoptosis and enhance the infection.

The current work demonstrates the significance of EphA2 which mediates the entry of *Ctr* into host cells. We, in addition, showed that the infection-induced ERK signaling is required for EphA2 upregulation and activation. The increased amount of EphA2 in infected cells was, however, not transported to the cell surface but rather gets recruited to the *Ctr* inclusion membrane where EphA2 interacts with pPI3K. We further demonstrated a crucial role of EphA2 for chlamydial development and the induction of apoptosis resistance in infected cells (Fig 11). Thus, EphA2 is involved in fundamental processes of chlamydial infection.

Similar to viruses, *Ctr* depends on the host cell for replication and survives only for a very limited time outside the host cells. We further show that EphA2 is also employed by *Ctr* to subvert host signaling for extended times after entry to ensure efficient development and protection of the host cell from apoptosis induction. Thus, engagement of cell surface receptors during the entry process may already pre-define the composition of inclusion-associated host proteins required for the propagation of *Ctr*.

The interaction of EB with purified EphA2 and the inhibition of this interaction with recombinant human Ephrin-A1 provide strong evidence that EB directly interact with EphA2. While EphA2 has been identified as receptor for hepatitis C virus [30] and Kaposi’s sarcoma-associated herpes virus [27] binding and entry, this is the first time EphA2 has been shown to function as a receptor for a bacterial pathogen. In line with a direct interaction, *Ctr* infection caused a strong activation of EphA2 within minutes, correlating with an unexpected strong increase in surface exposure of the receptor. Since the overall amount of EphA2 did not significantly increase until 3 h p.i., we can speculate that this rapid and strong surface display depended either on reduced receptor recycling or enhanced receptor exposure. Interestingly, at 3 h p.i., the total amount of EphA2 already started to increase and the elevated surface display was reduced to the level of uninfected cells and remained stable until the end of the infection cycle.

Our recent data suggested that *Ctr*-infected cells prevent re-infection to protect the intracellular niche (Karunakaran et al., submitted). The EphA2 overexpressing cells infected for 24 h were further re-infected with new EB for 4 h (re-infection assay). This assay increased 2.5-fold of new EB in EphA2 overexpressing infected cells compared to untransfected-infected neighboring cells (S5A–S5C Fig). Thus, preventing translocation of EphA2 to the cell surface may protect the intracellular niche as well as gaining EphA2 intracellular signals. It may also prevent contact of EphA2 with its ligand Ephrin-A1 on the surface of neighboring cells, which would reverse EphA2 signaling of the infected cell.

This profile of EphA2 surface display and receptor upregulation induced by chlamydial infection is strikingly different from that observed after Ephrin-A1 stimulation. EphA2 is rapidly endocytosed upon Ephrin-A1 ligation and degraded in lysosomes [42]. This so-called ligand-dependent signaling involves phosphorylation of EphA2 at Tyr594 [43] and interferes with the activation of the PI3K and MEK/ERK pathway [44]. The effect of *Ctr* infection on EphA2 is in line with ligand-independent signaling that causes EphA2 activation, supports PI3K/Akt activation and prevents EphA2 degradation. Activation of PI3K is, however, not completely dependent on activated EphA2 during chlamydial infection which indicates a possible role of other PI3K-inducing pathways. We found EphA2 activation not only at the cytoplasmic membrane during the initial phase of infection but also surrounding the endocytosed bacteria, indicating that this surface receptor continues to activate the PI3K pathway from within the infected cells. Signaling via EphA2 is thus not only important for the induction of bacterial entry but also required for maintaining a favorable niche for the replication of *Ctr*.

We show the recruitment of active EphA2 to the inclusion membrane during the mid-phase of the infection cycle indicating that EphA2-mediated signaling occurs at the cytosolic side of the inclusion membrane. We hypothesize that EphA2 initially remains associated with the endosomal membrane during bacterial entry. We currently do not know how the receptor is recruited to the inclusion at late infection time points and whether this requires secreted chlamydial ligands. Interestingly, in line with our findings, the activation of the PI3K pathway upon *Ctr*-serovar D infection has recently been demonstrated to depend on infection-induced RTK-EGFR phosphorylation [45]. Further, the EGFR co-localized with F-actin at the periphery of the inclusion [45]. These findings together with our results strengthen the significance of RTK’s functioning as intracellular signaling receptors at the inclusion besides being the cell surface receptor in *C*. *trachomatis* infection.

Several lines of evidence support a crucial role of EphA2 in PI3K activation during chlamydial infection. Both chemical inhibition of EphA2 kinase activity (S1E Fig and Fig 7) and of PI3K [13,14] prevented *Ctr* infection if the inhibitor was added either prior to or simultaneously with infection. These effects were milder if the inhibitors were applied after the EB to RB transition. We therefore postulate that early activation of the EphA2-PI3K pathway is important for chlamydial development. During this phase of infection, EphA2 levels remain largely unchanged indicating that post-translational modification by phosphorylation is the main mechanism of activation. During the middle and late phase of infection, the PI3K pathway appears to be less important since *Ctr* is able to develop irrespective of whether PI3K is active or not (see Figs 7 and 8A, [14]). The dose-dependent downregulation of mid phase-chlamydial infection by dasatinib, which can inhibit the activity of EphA2, Src, BCR-Abl, KIT and PDGFR [31,32], did not correlate well with the downregulation of the PI3K pathway (Fig 7). However, a kinase inactive EphA2 mutant also affected PI3K activation and *Ctr* infection, demonstrating a role of the EphA2 kinase activity in chlamydial infection. Clearly, the effect of the EphA2ΔIC (entire cytoplasmic deletion) was stronger suggesting that the cytoplasmic domain of EphA2 in addition to its kinase activity has an important function (Fig 6G). Apart from the PI3K, there are several other downstream targets of EphA2 like Ras-GTPase activating protein (RasGAP), Src, Abl family of non-receptor tryosine kinases and Rac1. During chlamydial infection, the Src kinase FYN has been shown to be essential for sphingomyelin trafficking to the *Ctr* inclusion [11]. Moreover, Rac1 activation is involved in chlamydial invasion into the host cells [46]. In addition, EphA2 is required for the association of Rab5 with internalized KSHV in the early macropinosome and productive trafficking of KSHV [35]. Further, Eph-EphA2 complexes are shown to be involved in various forms of endocytosis and cellular trafficking pathways [47]. Thus, the central role of EphA2 in cytoskeletal regulation and intracellular trafficking may be exploited by *Ctr* for efficient cell entry, replication and development.

We could demonstrate that EphA2 is required to maintain apoptosis resistance during *C*. *trachomatis* serovars L2 and D infection. This function very likely depends on the role of EphA2 in activation of the PI3K pathway, which plays a role in at least two anti-apoptotic signaling pathways activated by *Chlamydia* infection: the inactivation of the pro-apoptotic BH3-only protein Bad [13] and the stabilization of the anti-apoptotic Bcl-2 family member Mcl-1 [14]. It is possible that maintaining apoptosis resistance supports the integrity of the host cell during chlamydial replication and thus supports the infection, although direct experimental proof for this hypothesis is missing. The crucial role of EphA2 for chlamydial replication and its long-lasting upregulation in the course of infection may have another important consequence. EphA2 overexpression in the majority of ovarian cancer patients predicts poor clinical outcome connected to shorter overall survival [48]. Ovarian cancers are heterogeneous at the cellular and molecular levels, however, 90% of the ovarian cancers have epithelial histology and are believed to arise from surface epithelial cells, e.g. from cells lining the fallopian tubes [49]. *C*. *trachomatis* is known to infect and damage the human fallopian tube [50,51] and infection correlates with ovarian cancer [52,53]. Our findings of a strong EphA2 upregulation in primary fallopian tube epithelial cells suggests that targeting EphA2 and probably other Eph receptors or its co-receptors could offer a tool to control *Chlamydia*-induced ovarian cancer. *Ctr-*serovar D, cause genital and perinatal infections [54,55], also requires EphA2 to invade the cell and to prevent apoptosis induced by TNF-α similar to *C*. *trachomatis* serovar L2 (Fig 10). Thus, at least two different serovars of *C*. *trachomatis* appear to be capable of interacting with EphA2 to amplify signals crucial for infection.

The current concept on how *Chlamydia* modifies the host cell to support its development and replication assumes a central role of bacterial effector proteins that are secreted via the bacterial type three secretion system into the inclusion membrane or into cellular compartments. These bacterial effector proteins interact with host cellular proteins and subvert the host to a supportive niche for *Chlamydia*. We demonstrate here that the cell surface receptor EphA2 is activated within seconds after bacterial interaction and remains active after endocytosis to support chlamydial development. The continuous signaling of endocytosed EphA2 proposes a new concept of how *Ctr* manipulates its host cell by immediately engaging cell surface receptors and recruiting them to the inclusion for long-term. It is very likely that other receptors, most likely of the Eph-family, are used in a similar way to orchestrate host cell signaling right from the beginning of a *Ctr* infection. Further studies are crucial to identify the chlamydial factor interacting with EphA2 as well as to unravel the downstream targets of EphA2 upon *Chlamydia* infection, which benefits *Chlamydia*. It will be worthy to further unravel the complexity of inclusion-associated cell surface receptors that may offer new relevant targets for future anti-chlamydial therapies.

## Supporting Information

S1 Fig Cell surface EphA2 inhibition affects the invasion of *Ctr*-EB. (A) Representation for invasion assay: Cells transfected with siRNAs or preincubated with antibodies or inhibitors were infected with EB at 35°C for 4–6 h. (B) HeLa cells were transfected with siRNA against luciferase or EphA2 or PDI or EphA2 and PDI for 40 h at 37°C. The knockdown efficiency was monitored by WB analysis against total EphA2, PDI and GAPDH. (C) Transfected cells of B) were infected with *Ctr* (MOI 15–20) for 4 h at 35°C. The cells were immunostained against EB and Actin filaments. Number of EB invaded the cell were counted from 10 separate fields of view. Shown is the mean ± SD of three independent experiments normalized to siLuci-infected cells. *P<0.05, **P<0.01. Error bars show mean ± SD. (D) Fimb cells were pretreated with control IgG or N-terminal specific antibody against EphA2 for 1 h at 4°C and then infected with *Ctr* (MOI-50) for 4 h. Cells were immunostained as (C) and quantified by counting the invaded EB out of 30 different cells. Shown is the mean ± SD of two independent experiments normalized to IgG control. *P<0.05. Error bars show mean ± SD. (E) HUVEC cells pretreated with DMSO control and with 2.5 μM or 5 μM DA, respectively, for 1 h at 37°C were infected with *Ctr* (MOI-20) for 6 h. Cells were fixed and immunostained as (C). Invaded *Ctr* was counted randomly from 20 different cells under the microscope. Shown is the mean ± SD of three independent experiments normalized to DMSO-treated infected cells. **P<0.01. Error bars show mean ± SD. (D, E) Magnification is indicated in size bar.
(TIF)

S2 Fig EphA2 overexpression enhances the invasion rate of *Ctr*-GFP. (A, B) HeLa cells were left untransfected or transfected with empty-pcDNA3 or EphA2-pcDNA3 for 40 h followed by infection with *Ctr*-GFP (MOI-50) for 6 h. EphA2 expression (A) and invaded *Ctr*-GFP (B) were checked by FACS under permeabilised condition. The graph (B) shows the mean fluorescence value of the UN or infected cells under permeabilised condition compared to the respective controls. Shown is the mean ± SD of three experiments. ***P<0.001. Error bars show mean ± SD. (A, B) UN: uninfected, Iso: isotype and perm: permeabilised. (C) Empty-pcDNA3 or EphA2-pcDNA3 transfected HeLa cells were infected with *Ctr* for 4 h (MOI-20). Cells were fixed and immunostained for EphA2 (EphA2), Actin (Phalloidin) and *Ctr* (Hsp60). Arrows were drawn to indicate the co-localization of invaded *Ctr* with EphA2 (yellow). Magnification is indicated in size bar.
(TIF)

S3 Fig Viable *Ctr* infection induces the expression and activation of intracellular EphA2 in different cells. (A) Culture medium of the UN as well as time course (as indicated) *Ctr-*infected cells were collected and TCA precipitated. The precipitated lysates were subjected to WB analysis against total EphA2. (B) Cells were UN or infected with viable *Ctr* or heat-inactivated *Ctr* (HI) (65°C, 30 min) at MOI-100 for 15 min or with MOI-2 for 24 h. Lysed cells were immunoblotted against pEphA2 and Actin. The blot was stripped and reprobed for total EphA2. Increased levels of total EphA2 upon 15 min p.i. depend on the high MOI of 100 used in this experiment. (C, D, E) HeLa or HUVEC or Fimb cells were UN or infected with *Ctr* (MOI-2) for different time points and subjected to WB analysis to determine the expression of the proteins indicated.
(TIF)

S4 Fig Association of EphA2 with *Ctr* inclusion and generation of *Ctr*-pIncA-flag. (A) Cells were transfected with EphA2-pcDNA3 and left UN or infected with *Ctr* (MOI-1) for different times as indicated. Cells were immunostained against EphA2 (EphA2, red) and *Ctr* (Hsp60, green). (B) Cells transfected with EphA2-pcDNA3 were infected with *Ctr* (MOI-1) and immunostained against EphA2 (EphA2, red), *Ctr* (Hsp60, green) and DNA (Draq5, blue). *Ctr* inclusion of the untransfected cells (indicated with white arrows) were smaller than the inclusion of EphA2-transfected cells. (A, B) Magnification is indicated in size bar. (C) The vector map of pIncA::SW2 modified from the plasmid pGFP::SW2 [23] by replacing GFP:CAT with IncA-HA-Flag. (D) HeLa cells were infected with *Ctr* wild type (WT) or *Ctr*-pIncA-flag for 24 h (MOI-1). Cells were lysed and the indicated proteins were detected by immunoblotting after separation on a 17% SDS PAGE gel to separate the endogenous IncA from *Ctr*-pIncA-flag expressing recombinant IncA.
(TIF)

S5 Fig EphA2 overexpressed in infected cells allow re-infection in 24 h *Ctr*-infected cells. (A, B) Re-infection assay: Cells transfected with EphA2-pcDNA3 were infected with *Ctr* (MOI-1) for 24 h followed by re-infection using EB (MOI-15) for 4 h. Cells were washed thrice with PBS to remove the unbound bacteria and immunostained against EphA2 (EphA2, red), *Ctr* (Hsp60, green) and Actin filaments (Phalloidin, blue). Microscopic view was made focusing on the newly re-infected *Ctr* (arrows). Nearby untransfected and EphA2-transfected cells (red) were shown in the same image with zoomed in white boxes for better magnification of invaded bacteria in EphA2 transfected cells comparing to the untransfected cells. Arrows were drawn to indicate the newly adhered or invaded *Ctr*. Magnification is indicated in size bar. (C) The total number of re-infected bacteria (both adhered and invaded new EB) in EphA2 overexpressed and untransfected-infected cells were counted for maximum of 30 cells. Shown is the mean ± SD of three independent experiments normalized to untransfected-infected cells. **P<0.01. Error bars show mean ± SD.
(TIF)

S6 Fig EphA2 expression and activation in *C*. *pneumoniae* and *Ctr*-serovar A-infected HeLa cells. (A, B) HeLa cells were left UN or infected with *C*. *pneumoniae* or *Ctr*-serovar A, centrifuged at 910 x g for 30 min and allowed to infect for the indicated time points. The cells were harvested and subjected to WB analysis against pEphA2, Hsp60 and Actin. The blot was stripped and reprobed for total EphA2.
(TIF)
