# Supplementary figures and images for: EphrinA2 Receptor (EphA2) Is an Invasion and Intracellular Signaling Receptor for Chlamydia trachomatis
Source: PLoS Pathog. 2015 Apr 23;11(4):e1004846. doi: 10.1371/journal.ppat.1004846 (PMC4408118; doi:10.1371/journal.ppat.1004846)

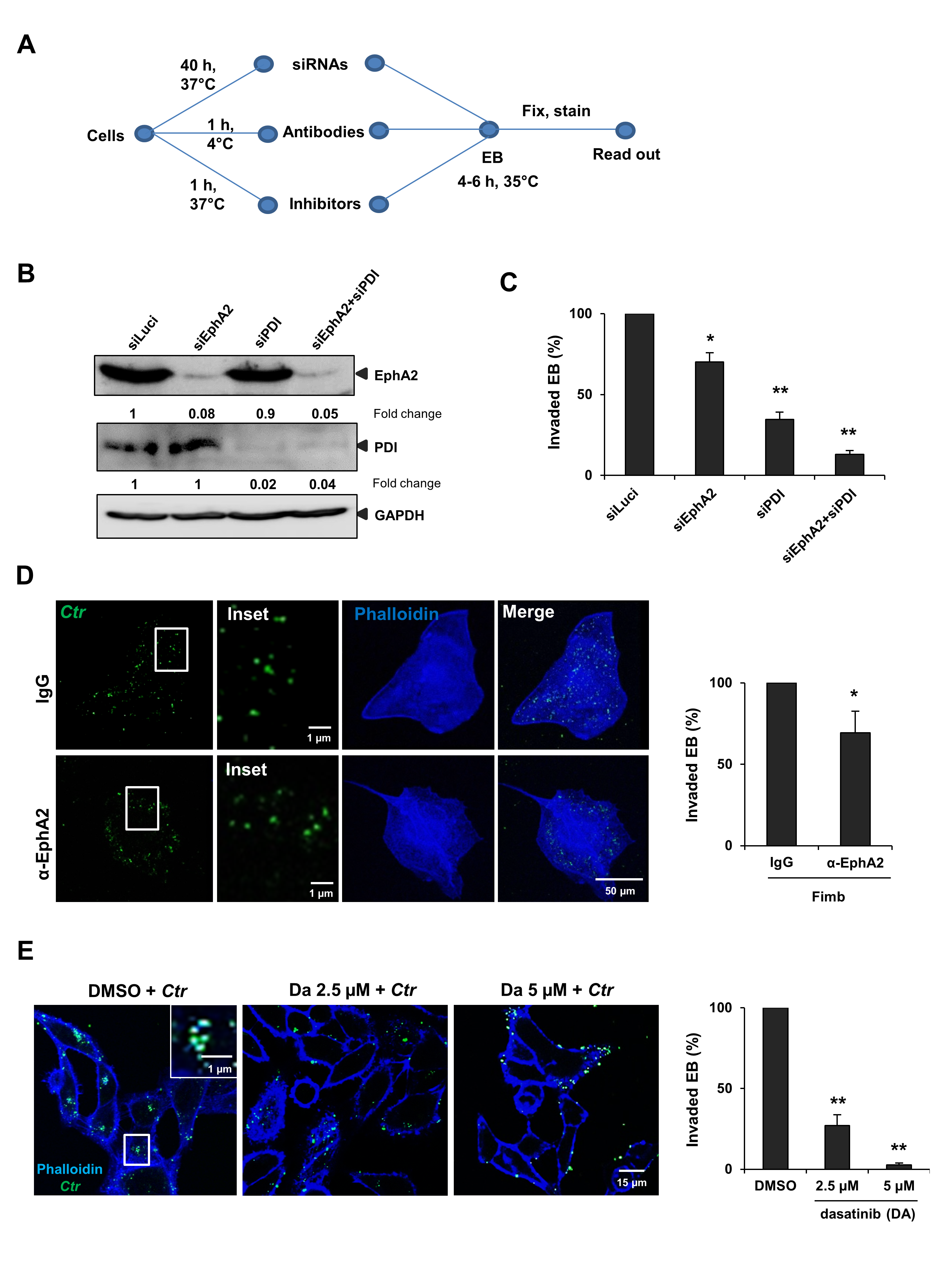

Supplement: S1 Fig — (A) Representation for invasion assay: Cells transfected with siRNAs or preincubated with antibodies or inhibitors were infected with EB at 35°C for 4–6 h. (B) HeLa cells were transfected with siRNA against luciferase or EphA2 or PDI or EphA2 and PDI for 40 h at 37°C. The knockdown efficiency was monitored by WB analysis against total EphA2, PDI and GAPDH. (C) Transfected cells of B) were infected with Ctr (MOI 15–20) for 4 h at 35°C. The cells were immunostained against EB and Actin filaments. Number of EB invaded the cell were counted from 10 separate fields of view. Shown is the mean ± SD of three independent experiments normalized to siLuci-infected cells. *P<0.05, **P<0.01. Error bars show mean ± SD. (D) Fimb cells were pretreated with control IgG or N-terminal specific antibody against EphA2 for 1 h at 4°C and then infected with Ctr (MOI-50) for 4 h. Cells were immunostained as (C) and quantified by counting the invaded EB out of 30 different cells. Shown is the mean ± SD of two independent experiments normalized to IgG control. *P<0.05. Error bars show mean ± SD. (E) HUVEC cells pretreated with DMSO control and with 2.5 μM or 5 μM DA, respectively, for 1 h at 37°C were infected with Ctr (MOI-20) for 6 h. Cells were fixed and immunostained as (C). Invaded Ctr was counted randomly from 20 different cells under the microscope. Shown is the mean ± SD of three independent experiments normalized to DMSO-treated infected cells. **P<0.01. Error bars show mean ± SD. (D, E) Magnification is indicated in size bar. (TIF) [file ppat.1004846.s001.TIF]

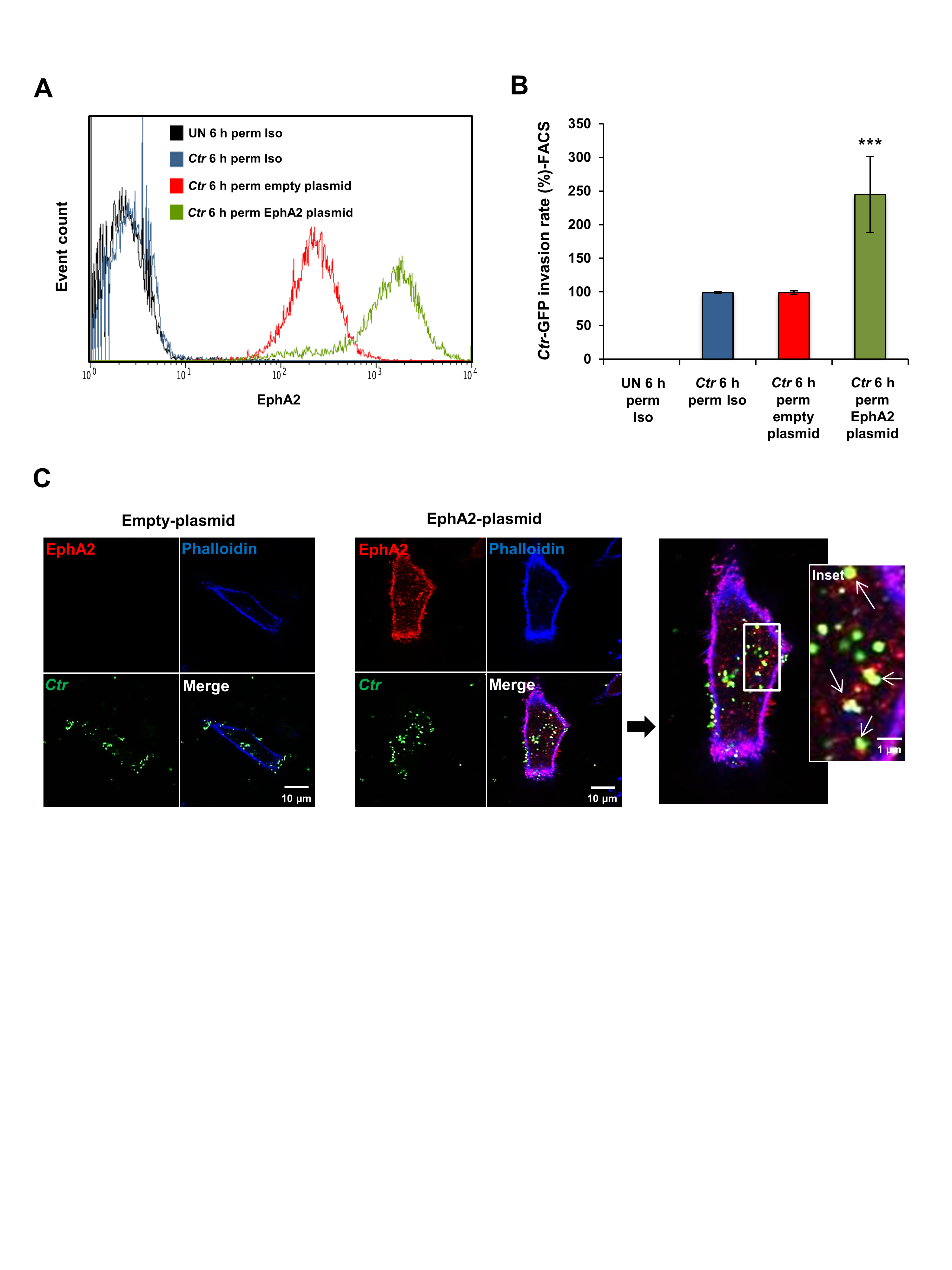

Supplement: S2 Fig — (A, B) HeLa cells were left untransfected or transfected with empty-pcDNA3 or EphA2-pcDNA3 for 40 h followed by infection with Ctr-GFP (MOI-50) for 6 h. EphA2 expression (A) and invaded Ctr-GFP (B) were checked by FACS under permeabilised condition. The graph (B) shows the mean fluorescence value of the UN or infected cells under permeabilised condition compared to the respective controls. Shown is the mean ± SD of three experiments. ***P<0.001. Error bars show mean ± SD. (A, B) UN: uninfected, Iso: isotype and perm: permeabilised. (C) Empty-pcDNA3 or EphA2-pcDNA3 transfected HeLa cells were infected with Ctr for 4 h (MOI-20). Cells were fixed and immunostained for EphA2 (EphA2), Actin (Phalloidin) and Ctr (Hsp60). Arrows were drawn to indicate the co-localization of invaded Ctr with EphA2 (yellow). Magnification is indicated in size bar. (TIF) [file ppat.1004846.s002.TIF]

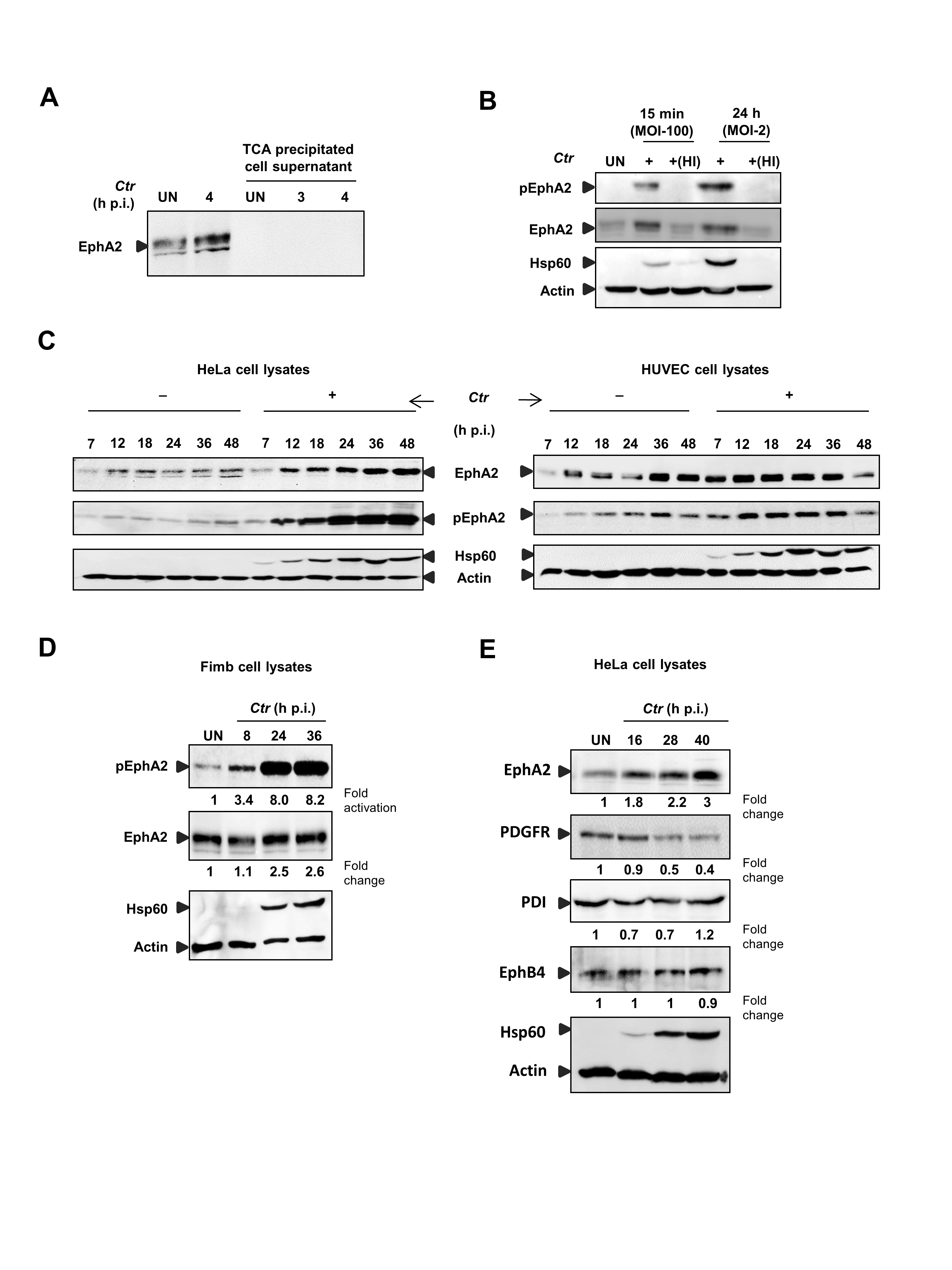

Supplement: S3 Fig — (A) Culture medium of the UN as well as time course (as indicated) Ctr-infected cells were collected and TCA precipitated. The precipitated lysates were subjected to WB analysis against total EphA2. (B) Cells were UN or infected with viable Ctr or heat-inactivated Ctr (HI) (65°C, 30 min) at MOI-100 for 15 min or with MOI-2 for 24 h. Lysed cells were immunoblotted against pEphA2 and Actin. The blot was stripped and reprobed for total EphA2. Increased levels of total EphA2 upon 15 min p.i. depend on the high MOI of 100 used in this experiment. (C, D, E) HeLa or HUVEC or Fimb cells were UN or infected with Ctr (MOI-2) for different time points and subjected to WB analysis to determine the expression of the proteins indicated. (TIF) [file ppat.1004846.s003.TIF]

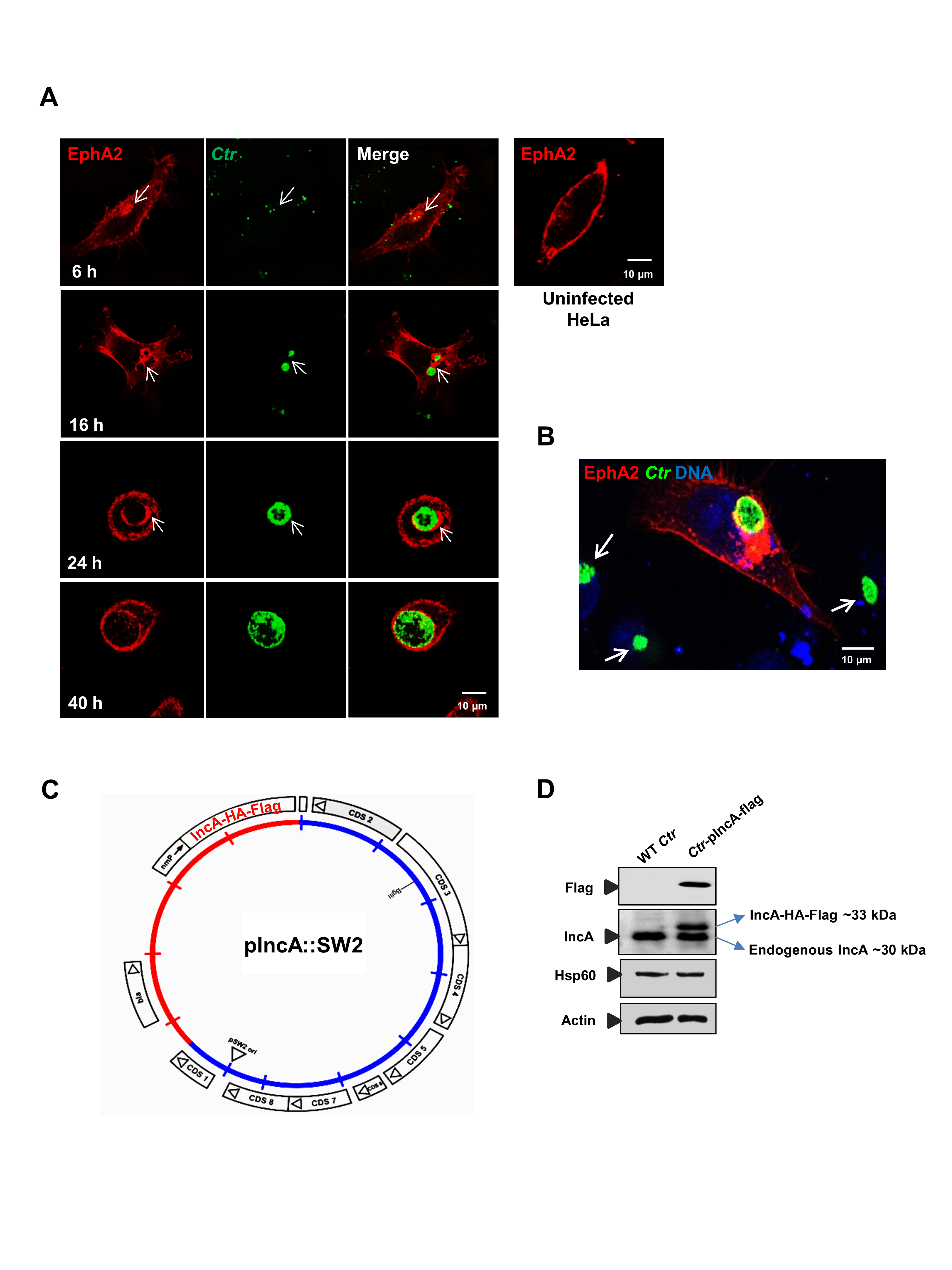

Supplement: S4 Fig — (A) Cells were transfected with EphA2-pcDNA3 and left UN or infected with Ctr (MOI-1) for different times as indicated. Cells were immunostained against EphA2 (EphA2, red) and Ctr (Hsp60, green). (B) Cells transfected with EphA2-pcDNA3 were infected with Ctr (MOI-1) and immunostained against EphA2 (EphA2, red), Ctr (Hsp60, green) and DNA (Draq5, blue). Ctr inclusion of the untransfected cells (indicated with white arrows) were smaller than the inclusion of EphA2-transfected cells. (A, B) Magnification is indicated in size bar. (C) The vector map of pIncA::SW2 modified from the plasmid pGFP::SW2 [23] by replacing GFP:CAT with IncA-HA-Flag. (D) HeLa cells were infected with Ctr wild type (WT) or Ctr-pIncA-flag for 24 h (MOI-1). Cells were lysed and the indicated proteins were detected by immunoblotting after separation on a 17% SDS PAGE gel to separate the endogenous IncA from Ctr-pIncA-flag expressing recombinant IncA. (TIF) [file ppat.1004846.s004.TIF]

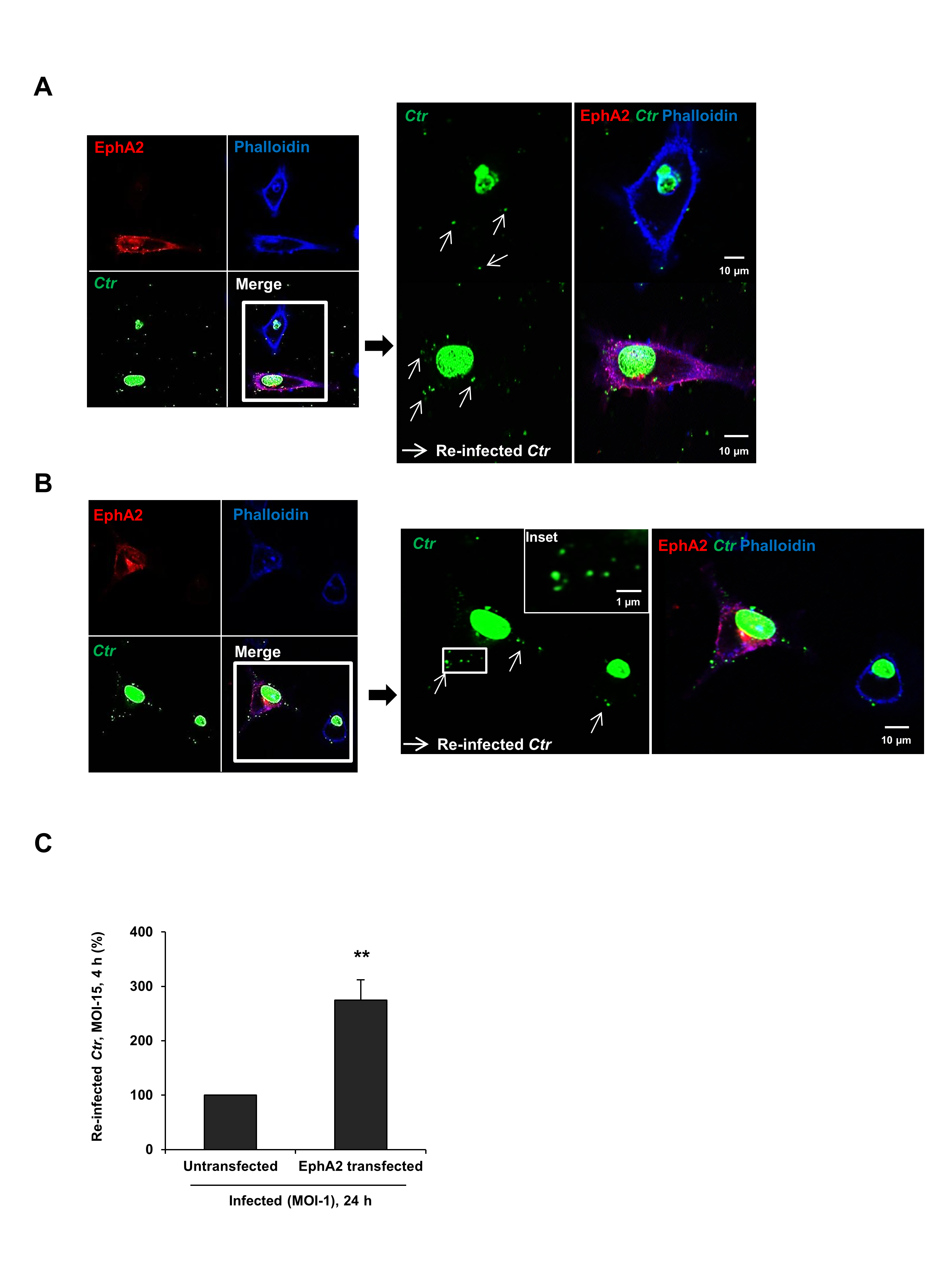

Supplement: S5 Fig — (A, B) Re-infection assay: Cells transfected with EphA2-pcDNA3 were infected with Ctr (MOI-1) for 24 h followed by re-infection using EB (MOI-15) for 4 h. Cells were washed thrice with PBS to remove the unbound bacteria and immunostained against EphA2 (EphA2, red), Ctr (Hsp60, green) and Actin filaments (Phalloidin, blue). Microscopic view was made focusing on the newly re-infected Ctr (arrows). Nearby untransfected and EphA2-transfected cells (red) were shown in the same image with zoomed in white boxes for better magnification of invaded bacteria in EphA2 transfected cells comparing to the untransfected cells. Arrows were drawn to indicate the newly adhered or invaded Ctr. Magnification is indicated in size bar. (C) The total number of re-infected bacteria (both adhered and invaded new EB) in EphA2 overexpressed and untransfected-infected cells were counted for maximum of 30 cells. Shown is the mean ± SD of three independent experiments normalized to untransfected-infected cells. **P<0.01. Error bars show mean ± SD. (TIF) [file ppat.1004846.s005.TIF]

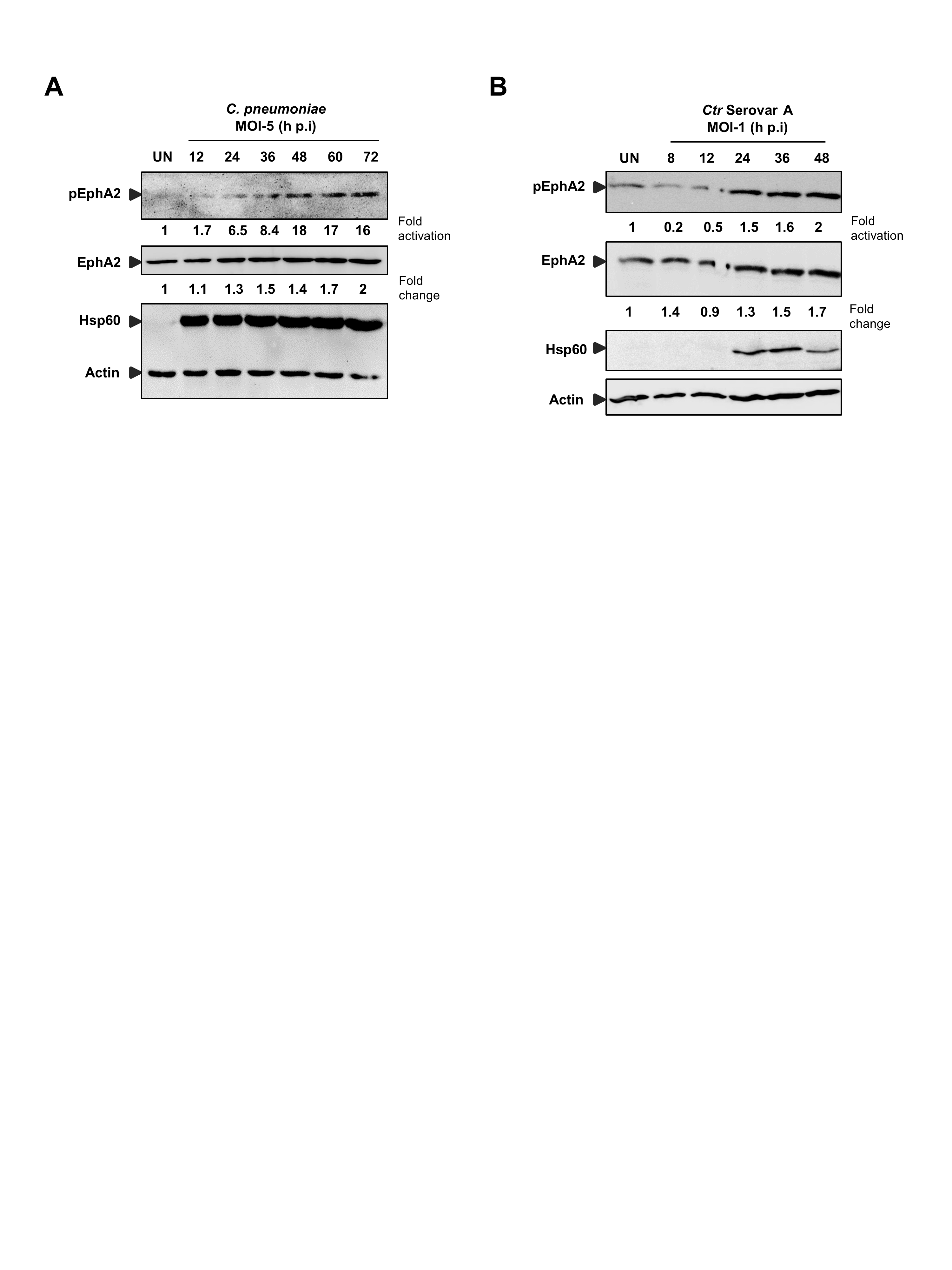

Supplement: S6 Fig — (A, B) HeLa cells were left UN or infected with C. pneumoniae or Ctr-serovar A, centrifuged at 910 x g for 30 min and allowed to infect for the indicated time points. The cells were harvested and subjected to WB analysis against pEphA2, Hsp60 and Actin. The blot was stripped and reprobed for total EphA2. (TIF) [file ppat.1004846.s006.TIF]
